# Predicting Brain Age Based on Spatial and Temporal Features of Human Brain Functional Networks

**DOI:** 10.3389/fnhum.2019.00062

**Published:** 2019-02-26

**Authors:** Jian Zhai, Ke Li

**Affiliations:** School of Mathematical Science, Zhejiang University, Hangzhou, China

**Keywords:** fMRI, resting state, functional connectivity, lifespan, predictive model, principal component

## Abstract

The organization of human brain networks can be measured by capturing correlated brain activity with functional MRI data. There have been a variety of studies showing that human functional connectivities undergo an age-related change over development. In the present study, we employed resting-state functional MRI data to construct functional network models. Principal component analysis was performed on the FC matrices across all the subjects to explore meaningful components especially correlated with age. Coefficients across the components, edge features after a newly proposed feature reduction method as well as temporal features based on fALFF, were extracted as predictor variables and three different regression models were learned to make prediction of brain age. We observed that individual's functional network architecture was shaped by intrinsic component, age-related component and other components and the predictive models extracted sufficient information to provide comparatively accurate predictions of brain age.

## Introduction

Senescence is an inevitable and complex biological process associated with brain changes. Moreover, there are notable individual differences in brain aging among the population and these differences might be an indication of deviation from healthy brain-aging trajectories for people suffering from developmental neuropsychiatric disorders such as Alzheimer's disease (AD) (Daffner, 2010; Koutsouleris and Sauer, 2013; Douaud et al., 2014). Thus a prediction of brain age for individuals could serve as a reliable biomarker to detect the risk of neurodegenerative diseases and be used for early diagnosis and therapy (Cole and Franke, 2017). For instance, predicted brain age being older than chronological age for a subject might imply accelerated brain aging arising from brain diseases.

Over the last three decades, functional magnetic resonance imaging (fMRI), especially resting-state functional connectivity fMRI (rs-fMRI) studies have significantly advanced our knowledge of human brain function and organization (Cole et al., 2014; Dubois, 2016; Dubois and Adolphs, 2016; Bassett and Sporns, 2017). Functional connectivity reflects the coherence between temporal fluctuations in the blood oxygen level dependent (BOLD) signal betweenconnectomics across two or more brain regions (Power et al., 2014; Liégeois et al., 2017). Increasing variety of studies have employed functional connectivity approaches to explore effects of aging on resting-state functional networks. A number of studies have revealed higher between-network connectivity and lower within-network connectivity in older adults compared with younger adults (Kobuti and Busatto, 2013; Chan et al., 2014; Yang et al., 2014; Grady et al., 2016; Spreng et al., 2016; Petrican et al., 2017; Zuo et al., 2017). For example, Spreng et al. (2016) observed reduced within-network and increased between-network functional connectivity (FC) across the default mode and dorsal attention networks. A measure of network segregation was defined to summarize values of within-network connectivity in relation to between-network connectivity in Chan et al. (2014) and they found that increasing age was accompanied by decreasing segregation of brain networks, which was consistent with finding of less within-network and more between-network connectivity with older age. Grady et al. (2016) revealed frontoparietal network (FPN) served as a switch to influence the age differences in default mode network (DMN) besides a similar finding with weaker within-network connectivity and stronger between-network connectivity. Apart from linear developmental patterns with aging (Onoda et al., 2012; Kobuti and Busatto, 2013; Chan et al., 2014; Petrican et al., 2017), quadratic lifespan patterns of development have been found to fit the development strategy better in some studies (Betzel et al., 2014; Cao et al., 2014; Douaud et al., 2014). For example, Betzel et al. (2014) found that FC in some brain areas showed an inverted U-shape pattern of an increase in connectivity during development and early adulthood and a decrease in older adults.

Even that human brain structure and function greatly vary across individuals has been recognized for several years (van Horn et al., 2008), researchers did not show widespread interest in personalized investigation of brain function until recent year, benefiting from technological advances and large brain datasets (Nooner et al., 2012; Van Essen et al., 2013; Finn et al., 2015; Xia and He, 2017). For example, a model was built from whole-brain functional network to predict sustained attention of individuals (Rosenberg et al., 2016), proving that functional connectivity pattern provided reliable measures of individual differences in behavior. Thus, there have been some studies trying to predict brain age for individuals through brain network approaches. By employing structural or functional neuroimaging data and machine learning methods, predictive models are learned on training datasets to make predicted brain age compared to chronological age based upon extracting different features (Wang et al., 2012; Mwangi et al., 2013; Han et al., 2014; Lin et al., 2015; Luders et al., 2016; Cole et al., 2017; Liem et al., 2017; Lancaster et al., 2018; Li et al., 2018). For example, Lin et al. (2015) employed artificial neural network to predict brain age based on structural connectivity network. Lancaster et al. (2018) used T1-weighted MRI scans for age prediction and Bayesian optimization method was adopted to optimize resampling parameters and improve prediction performance. Compared with studies with prediction frameworks based on structural features, there have been less studies predicting brain age built on functional connectivity features. For instance, Dosenbach et al. (2010) adopted a multivariate pattern analysis (MVPA) tool to construct a biomarker from functional connectivity with which accurate predictions about individuals' brain maturity across development were made.

Base on the above findings, we hypothesized that sufficient information would be extracted from resting state fMRI data to make accurate predictions of brain age. In the current study, two publicly available Enhanced Nathan Kline Institute—Rockland Sample (NKI-RS-E) dataset (aged 6 to 85 years) and Nathan Kline Institute—Rockland Sample (NKI-RS) dataset were employed. Resting-state functional network was constructed for each subject. Principal component analysis was performed at the network-based level across all subjects and components acquired were probed subsequently to explore whether they were meaningful and their relationships with age. Then coefficients across the components were extracted as predictor variables and different regression models were learned to make prediction of brain age. What's more, two other feature extraction/reduction methods, edge-based method and temporal features based method, were also proposed as a control and complementary analysis, and we expected that accurate prediction could be achieved. Finally, three distinct regression models were trained, K-fold cross-validation was performed on NKI-RS-E dataset and external validation was performed on NKI-RS dataset.

## Materials and Methods

### Subjects and Imaging Protocols

In this work, we used two datasets: Enhanced Nathan Kline Institute—Rockland Sample (NKI-RS-E) data and Nathan Kline Institute—Rockland Sample data, for internal and external validation. Characteristics of subjects for two datasets are shown in Supplementary Tables 1, 2.

#### Enhanced Nathan Kline Institute—Rockland Sample (NKI-RS-E) Data

Multiband resting-state fMRI (R-fMRI) data were acquired from the publicly available Enhanced Nathan Kline Institute—Rockland Sample (Nooner et al., 2012) which is an ongoing, institutionally centered endeavor aimed at creating a large-scale (*N* > 1000) community sample of participants across the lifespan. We selected data of 496 individuals from this dataset (304 females; mean age: 40.8; range 6 and 85). Institutional Review Board Approval was obtained for this Project at the Nathan Kline Institute(Phase I #226781 and Phase II #239708) and at Montclair State University(Phase I #000983A and Phase II #000983 B).Written informed consent was obtained for all study participants. Written consent and assent was also obtained from minor/child participants and their legal guardian.

MRI data were collected in a 3T Siemens TIM Trio scanner. Resting fMRI data were acquired using multiband EPI with the following parameters: voxel size = 3 × 3 × 3 mm^3^; matrix = 74 × 74; field of view = 222 mm; TR = 645 ms; TE = 30 ms; 900 volumes and 40 axial slices. For spatial normalization, an MPRAGE image was acquired. During the resting state data acquisition, each participant was instructed to simply rest with eyes open. Eleven participants were excluded from analysis for missing time points or being unable to decompress datasets.

#### Nathan Kline Institute—Rockland Sample (NKI-RS) Data

This dataset consisted of 207 subjects between the ages of 4 and 85 year-old (mean age: 35.5). All subjects underwent imaging scans in a 3T Siemens TIM Trio scanner. Images were acquired using an EPI sequence with the following parameters: TR = 2500 ms; TE = 30 ms; voxel size = 3 × 3 × 3 *mm*^3^; matrix = 74 × 74; field of view = 216 mm; 260 volumes and 38 axial slices. A high resolution MPRAGE image was acquired for each subject. Six participants were excluded from analysis for missing time points or being unable to decompress datasets.

### Data Preprocessing

The Data Processing Assistant for Resting-State fMRI [DPARSF, (Chao-Gan and Yu-Feng, 2010; Yan et al., 2016)] was used for preprocessing for both datasets. The first 10 volumes were removed for signal equilibrium. Then the slice timing was done for correcting image acquisition time differences. Head motion correction was carried out for each subject and subjects with mean FD > 0.2 mm were discarded (Jenkinson et al., 2002). As a result, 10 subjects were excluded for further analysis in NKI-RS-E data sets and 28 subjects were excluded from NKI-RS data sets. MPRAGE images were co-registered to the mean functional image after realignment. The transformed structural images were then segmented into gray matter, white matter and cerebrospinal fluid (Ashburner and Friston, 2005). Normalization from individual native space to MNI space was done by the DARTEL tool (Ashburner, 2007). Nuisance covariates (Friston et al., 1996) (including 6 head motion parameters, 6 head motion parameters one time point before, and the 12 corresponding squared items) were regressed from the data. Global signal regression (GSR) was not performed here. Data were spatially smoothed with a Gaussian kernel (FWHM = 4 mm). Before the temporal filtering was finally performed, fractional amplitude of low frequency fluctuations (fALFF) was computed using the timecourses for each voxel. At last, temporal band-pass filtering (0.01–0.1 Hz) was carried out to reduce the influence of low-frequency drifts and the high-frequency physiological noises. Thus, the resulting time courses were used for the brain network construction and later analysis.

### Network Construction

Power-264 template (Power et al., 2011) was employed to generate 264 nodal ROIs, and each node denoted 5-mm radius spheres centered on previously reported coordinates. This template were defined based on meta-analysis and functional connectivity mapping, and has been widely used in recent years. We obtained average time course for each node from the preprocessed data. Pearson's correlation coefficients between each pair of nodes were calculated using the time course obtained above and normalized to *z* scores using the Fisher transformation to improve normality (Fisher, 1915),

$$\begin{array}{l} z\;=\;\frac{1}{2}\ln\left(\frac{1+r}{1-r}\right) \end{array}$$

which generated a 264 × 264 symmetric correlation matrix *M* (functional connectivity matrix) for each participant. For a control analysis, we also used another different but also widely used brain template, consisting of 160 ROIs which was employed by Dosenbach et al. (2010).

### Principal Component Analysis and Relationship With Age

In this section, we employed principal component analysis on the matrices of all participants. However, we used this method to cope with the data in a quite different way. For functional connectivity matrix of each subject, we took the upper triangle of it (excluding self-connections and redundant connections) and acquired a connectivity matrix ***D*** (*m* × *n*, *n* is the number of subjects, m is the length of the triangle vector) across all subjects. Then we did the principal component analysis on the raw data ***D***.

T = D · P

where **P** is the matrix (*n* × *n*) of weights whose columns are the eigenvectors of **D**^**T**^**D** and **T** is the matrix (*m* × *n*) of principal components whose columns correspond to different components. Principal components (PC) in **T** are mutually orthogonal and extracted in decreasing order of importance, in which the lower-order principal components account for most of the variance in the original data. Connectivity vector ***D*** can be reconstructed by

$$D=T\;\cdot \;P^{T}$$

If the first *L* PCs were kept and the left components which were less important are ignored, we got the truncated transformation of above formula

$$D\approx T_{L}\;\cdot \;P_{L}^{T}$$

where the matrix **T**_**L**_ has *m* rows and *L* columns, and ${\text{P}}_{\text{L}}^{\text{T}}$ keeps *L* rows and *n* columns. For *i*th column of **D** (the triangle vector of participant *i*),

$$\begin{array}{l} D_{i}=\overset{L}{\underset{j=1}{\sum }}p_{ij}T_{j} \end{array}$$

where *T*_*j*_ is the *j*th PC (i.e., *j*th columns of matrix **T**_**L**_) and *p*_*ij*_ is the coefficient. Moreover, we could transform *T*_*j*_ to a matrix by setting it to be the triangle of a symmetric matrix, in which the diagonal entries are set to be 1. The transformed matrix of the *i*th PC was represented by*PC*_*j*_. Therefore, the functional connectivity matrix *M*_*i*_ could be expressed by

$$\begin{array}{l} M_{i}=p_{i1}PC_{1}+p_{i2}PC_{2}+\ldots +p_{iL}PC_{L} \end{array}$$

We also defined the multi-subject matrix by calculating correlations across the concatenated time series of all subjects as the intrinsic resting-state network. Further we computed the correlation between age and functional connectivity for each ROI-pair to acquire a matrix of age effects on functional connectivity. We compared these two matrices to the components we had already obtained.

### Functional Connectivity Across the Lifespan

In addition to considering functional connectivity for each edge, we also focused on ten 10 consistently identified networks in Power et al. (2011): SMN, sensorimotor network; CON, cingulo-opercular network; Aud, auditory network; DMN, default mode network; Vis, visual network; FPN, fronto-parietal network; SN, salience network; Sub, subcortical network; VAN, ventral attention network; DAN, dorsal attention network. Within-network connectivity, which was represented by expressions like Within (DMN), was calculated as mean *z*-value of all ROI-ROI pairs within that network. Between-network connectivity, e.g., Between (DMN, SMN), was acquired by calculating the mean *z*-value between each node of a network and all other nodes of all other networks likewise. We further calculated the difference between within-network connectivity for one network and between-network connectivity that connected it with all other 9 networks, which was given by expressions like Within-Between(DMN).

To explore the functional connectivity linear or quadratic changes at both the edge-level and network-level across the lifespan, two linear regress models were utilized with sex, motion, and brain volume as covariates, which could be formulated as follows:

$$\begin{array}{l} FC=\;\beta_{0}+\beta_{1}\times age+\;\beta_{2}\times sex+\beta_{3}\times FD\\ \;+\beta_{4}\times TIV+\varepsilon ,\\ FC=\;\beta_{0}\;+\beta_{1}\times age+\;\beta_{2}\times age^{2}+\beta_{3}\times sex+\beta_{4}\times FD\\ \;+\beta_{5}\times TIV+\varepsilon . \end{array}$$

FC could denote connectivity for edge, within-network, between-network or the within-between. FD, which was included to control for the residual effect of head motion, denoted the mean frame-wise displacement. TIV denoted total intracranial volume for each subject. Sex was modeled as 0–1 covariate and ε was modeled as the random error. The *p*-value for the regression model in both equations above were needed to be significant in order to identify the linear or quadratic relationship between age and each functional connectivity. The T-statistic was used to measure the significance and *p*-values for both linear and quadratic models were FDR corrected.

### Temporal Measures Across the Lifespan

fALFF (Zou et al., 2008) is the ratio of power spectrum of low-frequency (0.01–0.1 Hz) to that of the entire frequency range (0.01–0.25 Hz). We firstly probed the correlations with age for the fALFF values of each voxel. Then average values of fALFF for each node within the Power-264 template were extracted and explored their linear or quadratic relationships with age likewise. The p-values for all the models were FDR corrected for multiple comparisons.

### Feature Selection/Reduction Method

There are always two stages to create a predictive model (Pereira et al., 2009; Brown and Hamarneh, 2016): firstly feature selection/extraction and reduction is performed, and then the regression model for continuous variables or classification model for discrete variables would be constructed. A variety of studies have shown that reducing the number of features can not only speed up computation, but also improve predictive performance (De Martino et al., 2008; Pereira et al., 2009; Esterman et al., 2010). Based on the sections above, three different methods were proposed in this feature extraction/selection section: network-based method, edge-based method and temporal feature method. For the network-based feature selection method, as functional connectivity matrix for each subject could be expressed by a combination of principal components, the coefficients across the components were extracted as predictor variables and regression models was learned later to make prediction of chronological age. As for temporal features extraction method, average values of fALFF for each node within the Power-264 template were used as feature for age prediction.

With regards to edge-based feature selection and reduction method, the functional connectivity for each ROI pair was treated as feature. What we needed to do would be to transform the high-dimensional FC space into a lower-dimensional FC space. A new feature reduction method was defined and employed here. Define *X* as the transposed matrix of ***D*** (**D** is defined as in section Principal Component Analysis and Relationship With Age) and *Y* (*n* × 1 vector, *n is the number of subjects*) as response vector representing age of the subjects. Feature reduction method is described as bellows:

(1) At first, both *X* and *Y* are standardized, where *X* (*n* × *m*, *n is the number of subjects, m* is the length of the triangle vector) is the connectivity matrix and *Y* (*n* × 1) is the age vector.
(2) Assume that *u*_1_ = *Xp*_1_ and maximize *Cov*(*u*_1_, *Y*), s.t., ||*p*_1_|| = 1, where *p*_1_ is the loading vector and *u*_1_ is the component vector. In this step, we get the component *u*_1_ by maximizing covariance between *u*_1_ and *Y*, which combines variance of *X* maximization and correlation with *Y* maximization simultaneously.
(3) Then do regression analysis, $X=u_{1}c_{1}^{T}+E$, *Y* = *u*_1_*r*_1_ + *F*, where *E* is residual matrix, *F* is residual vector, *c*_1_ is the projection vector on component *u*_1_ for connectivity matrix *X* and *r*_1_ is the projection value on *u*_1_ for age vector *Y*.
(4) Regard *E* as the new *X* and *F* as the new *Y*, do analysis as above iteratively until the first *l* loading vectors *p*_1_, *p*_2_, …, *p*_*l*_ and first *l* component vectors *u*_1_, *u*_2_, …, *u*_*l*_ are acquired.
(5) Define *P* to be the loading matrix with columns *p*_1_, *p*_2_, …, *p*_*l*_ and *U* to be the component matrix with columns *u*_1_, *u*_2_, …, *u*_*l*_. Apparently, *U* is the feature matrix to be used as predictor variables after feature reduction and the dimensionality of edge feature is reduced to *l* from *m*.

### Prediction Model of Chronological Age

Based on the three feature selection/reduction methods described above, three different predictive models, ordinary linear regression (i.e., OLS regression), support vector regression (SVR) and Least absolute shrinkage and selection operator (Lasso) regression were chosen in the present work and the prediction performance was compared between all these predictive models. Assume that the feature space $X=\left\{x_{i}\;:x_{i}\;\in \;{\mathbb{R}}^{d}\right\}$ is a real vector space with dimensionality *d* after the feature selection/reduction methods have been conducted. For any regression models, what we need to do is to determine the parameters for the basic regression function via minimizing the corresponding loss function:

$$y_{i}=\beta \;\cdot \;x_{i\;}+\beta_{0}$$

where parameters *β*_0_ and *β* are a scalar and a vector respectively and *β* ∈ ℝ^*d*^.

For ordinary linear regression, the loss function has the following form:

$$L\left(y,\;x\right)=\sum_{i=1}^{n}{\left(y_{i}-\beta_{0}-\beta \;\cdot \;x_{i\;}\right)}^{2}$$

where *n* is the number of feature vectors. The method of OLS is a classical method for parameter estimation and provides minimum-variance mean-unbiased estimation. However, when number of predictor variables is much higher than the number of observations or there exists multicollinearity among the predictor variables, the least Square estimates might become unreliable.

Lasso (Tibshirani, 1996) is a regression analysis method which can perform both variable selection and regularization in order to enhance the prediction accuracy and interpretability. The basic loss function for Lasso method is as follows:

$$L\left(y,\;x\right)=\frac{1}{2n}\sum_{i=1}^{n}{\left(y_{i}-\beta_{0}-\beta \;\cdot \;x_{i\;}\right)}^{2}+\lambda \;\sum_{j=1}^{p}\;\left|\beta_{j}\right|)$$

where λ is a nonnegative regularization parameter. L1 penalty is introduced in Lasso compared with OLS method, thus forcing certain coefficients to be set to zero and effectively choosing a simpler model.

As to SVR, it is an extension of the classical SVM method and has been widely used in neuroscience research (Ben-Hur et al., 2008; De Martino et al., 2008; Bray, 2009; Esterman et al., 2009; Dosenbach et al., 2010; Rubinov and Sporns, 2010; Ullman et al., 2014). Its loss function is described as follows:

$$L\left(y,\;x\right)=\frac{1}{2}||\beta {||}^{2}+C\sum_{i=1}^{n}\;l_{\epsilon }\left(y_{i}-\beta_{0}-\beta \;\cdot \;x_{i\;}\right)$$

where $l_{\epsilon }\;(z)=\;\{\begin{array}{c} 0,\;if\;|z|\;\leq \;\epsilon ;\;\\ |z|-\;\epsilon ,\;otherwise.\; \end{array}\;$, L2 penalty and constant *C* are introduced to trade off the empirical risk and model complexity.

The three regression methods were conducted to learn the predictive model, NKI-RS-E dataset was used as the internal dataset and *K*-fold cross-validation was performed (*K* = 10). During *K*-fold, each fold was designated as the test samples in turns while the remaining *k*−1 folds were used to train the predictive model. The model learned from the training samples was then used to make a real-valued prediction about the test samples. Predictive models trained in the entire NKI-RS-E dataset were applied to NKI-RS dataset for external validation.

After *K*-fold cross-validation had been completed, the accuracies for all *K*-fold rounds were averaged together to generate the prediction accuracy of internal validation. Both the external and internal accuracies were reported. The prediction performance was quantitatively evaluated by two statistical criterions: Pearson correlation coefficient *r* and mean absolute error (MAE). Pearson correlation coefficient is a measure of the linear correlation between two variables X and Y and has been used to evaluate predictions of a continuous variable in a variety of fMRI literature (Ullman et al., 2014; Shen et al., 2017). In our research, Pearson correlation coefficient *r* was used to measure the strength of linear correlation between chronological age and predicted brain age. Larger Pearson correlation coefficient *r* means better predictive performance. As for the MAE, it was adopted to measure the average magnitude of the residuals between chronological ages and predicted ages. Both statistical criterions are described as follows:

$$\begin{array}{l} r=\frac{\sum_{i=1}^{n}\left(x_{i}-\bar{x}\right)\left(y_{i}-\bar{y}\right)}{\sqrt{{\sum_{i=1}^{n}\left(x_{i}-\bar{x}\right)}^{2}}\sqrt{\sum_{i=1}^{n}{\left(y_{i}-\bar{y}\right)}^{2}}}\\ \;MAE=\frac{\sum_{i=1}^{n}\left|x_{i}-y_{i}\right|}{n} \end{array}$$

where *x*_*i*_ is the chronological age, *y*_*i*_ is the predicted brain age, $\overset{\bar{}}{x}$ is the average of chronological ages across the subjects, $\overset{\bar{}}{y}$ is the average of predicted ages and *n* is the number of subjects in the dataset.

### Control Analysis for Predictive Model

On one hand, we utilized the method proposed in Dosenbach et al. (2010) to our datasets and made a comparison with our models in prediction results. On the other hand, to investigate the effect of brain parcellation on the prediction performance, another different but widely used brain template (Dosenbach et al., 2010) was employed, in which feature selection/reduction methods and regression models in our study were adopted. The prediction performance based on different templates was also compared.

### Significant Edges and Networks in Predictive Model

In order to explore which edges or networks drive accurate prediction in our predictive models, we performed 10,000 permutations to probe edges with statistically significant weights in the three regression models combined with edge-based feature selection/reduction method. Ages were randomly shuffled across subjects in each permutation, i.e., assigning ages to different subjects to break the true brain–age relationship, and then different regression models were learned. Beta coefficients of the regression models for each edge were obtained in any permutation. The distribution of the test statistic was acquired from the permutations and significance for each edge was determined by whether its real beta value differed from the empirical distribution (two-tailed *p* < 0.001).

## Results

### Principal Component Analysis and Relationship With Age

**Figure 1:**
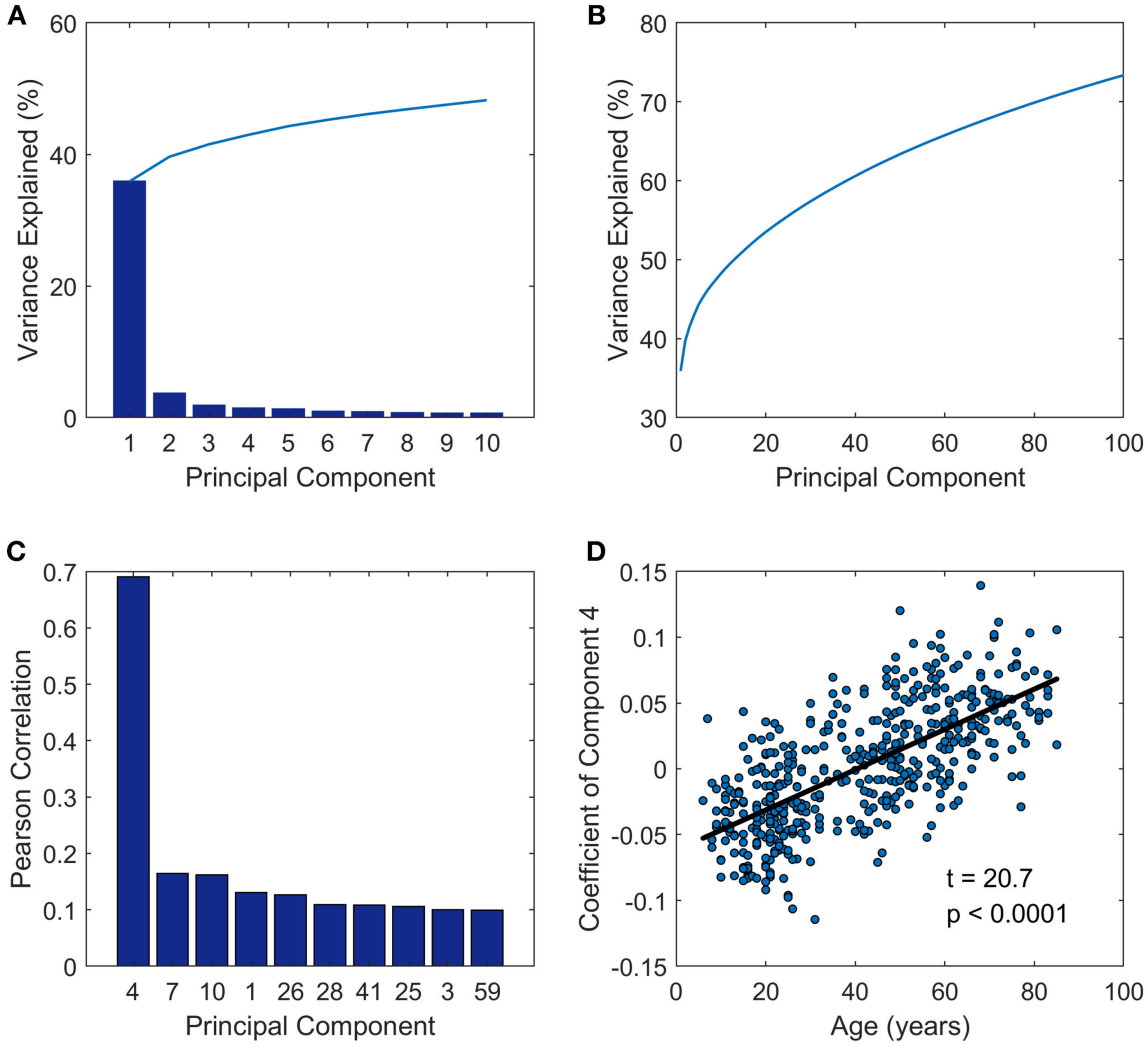
Principal component analysis results. **(A)** Variance explained by the first 10 components. **(B)** Variance explained by the first 100 components. **(C)** The first ten components which were most closely correlated with age. **(D)** The coefficients of PC 4 across subjects (y-axis) vs. ages of subjects (x-axis) fitted by a linear regression model.

The principal component analysis was performed on the FC matrices across all the subjects. As we can see in Figure 1, the first PC of the analysis accounted for approximately 36%, and PC 2, PC 3, PC 4, and PC 5 accounts for 3.7, 1.9, 1.4, and 1.3% of the inter-subject FC matrix variance, respectively (Figure 1A). The first 100 PCs accounted for more than 70% of the total variance (Figure 1B). Next we investigated the relationships between PC and age by calculating the Pearson correlation between the coefficients of each PC and age vector (Figure 1C), and observed that the PC coefficients of the fourth PC had a correlation coefficient as high as 0.69 to age vector. Then a linear regression model was applied further to probe the relationship between age and coefficient of PC 4 across subjects (Figure 1D). Adolescents and young people under age 40 have negative coefficient, while older people aged over 40 tend to have positive coefficient of PC 4.

**Figure 2:**
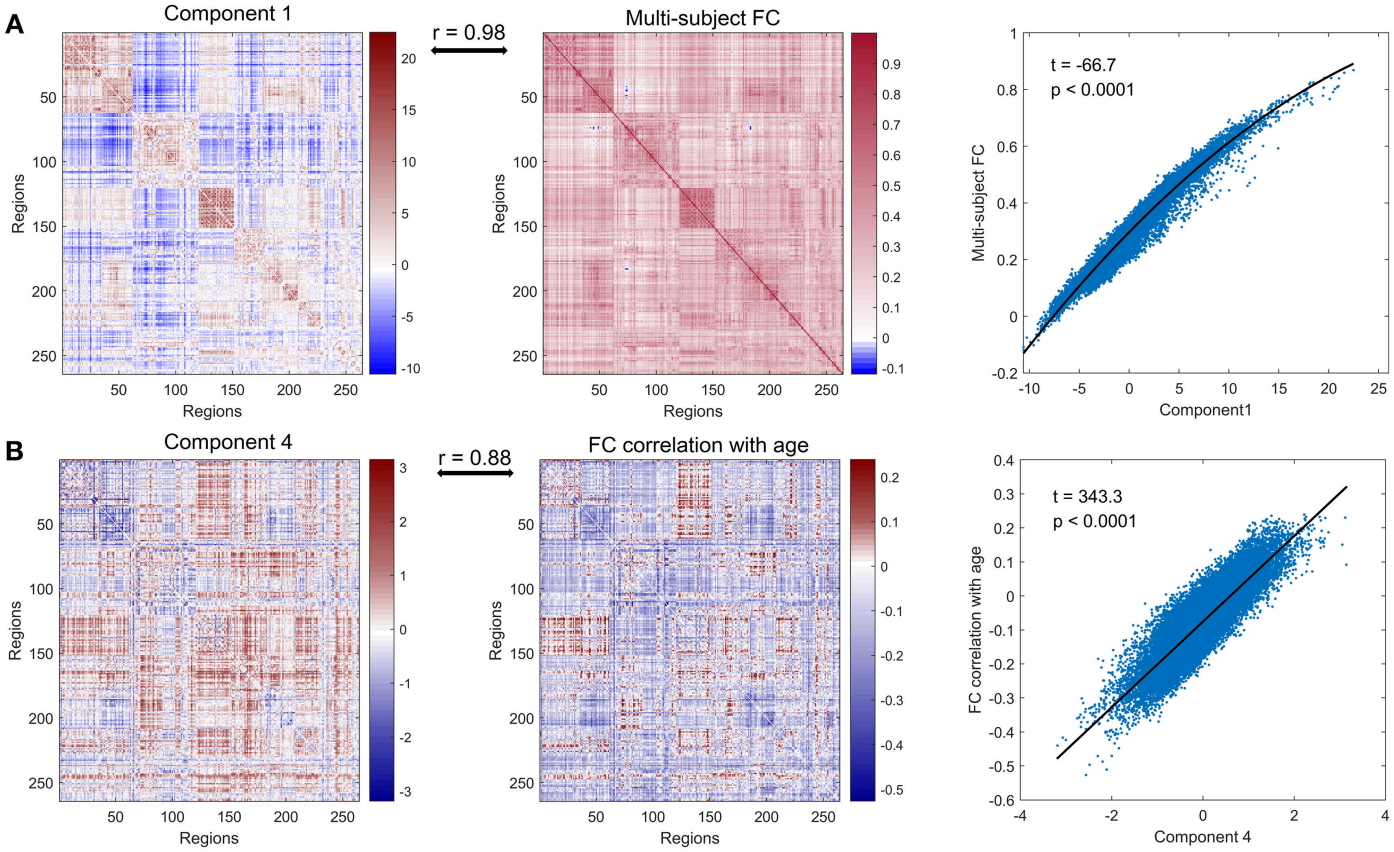
Further analysis of principal component 1 and principal component 4. **(A)** PC 1 (left panel), multi-subject matrix by calculating correlations across the concatenated time series of all subjects (middle panel) and the quadratic association between values of PC 1 (x-axis) and values of multi-subject matrix (y-axis) (right panel). PC 1 and multi-subject matrix are highly correlated (*r* = 0.98). **(B)** PC 4 (left panel), the matrix of age effects on edges by computing the correlation between age and functional connectivity for each edge (middle panel) and the linear association between values of PC 4 (x-axis) and values of age-effect matrix (y-axis) (right panel). PC 4 and age-effect matrix are highly correlated (*r* = 0.88).

We defined the multi-subject FC matrix by calculating correlations across the concatenated time series of all subjects and found that the multi-subject matrix and the first principal component were highly similar (Figure 2A, *r* = 0.98, *p* < 0.0001). Then a regression model was applied to explore the relationship between PC 1 and multi-subject matrix, and connectivity of edges in multi-subject matrix could be accurately fitted by values of edges in PC 1 using a quadratic regression model (Figure 2A, *t* = −66.7, *p* < 0.0001). To further explore the correlation of the PC 4 to age, we acquired a matrix of age effects on functional connectivity by computing the correlation between age and functional connectivity for each ROI-pair. As we expected, the PC 4 and the age-effect matrix were highly similar (Figure 2B, *r* = 0.88, *p* < 0.0001). Values of age-effect matrix were fitted well by values of PC 4 using a linear regression model (Figure 2B, *t* = 343.3, *p* < 0.0001).

**Figure 3:**
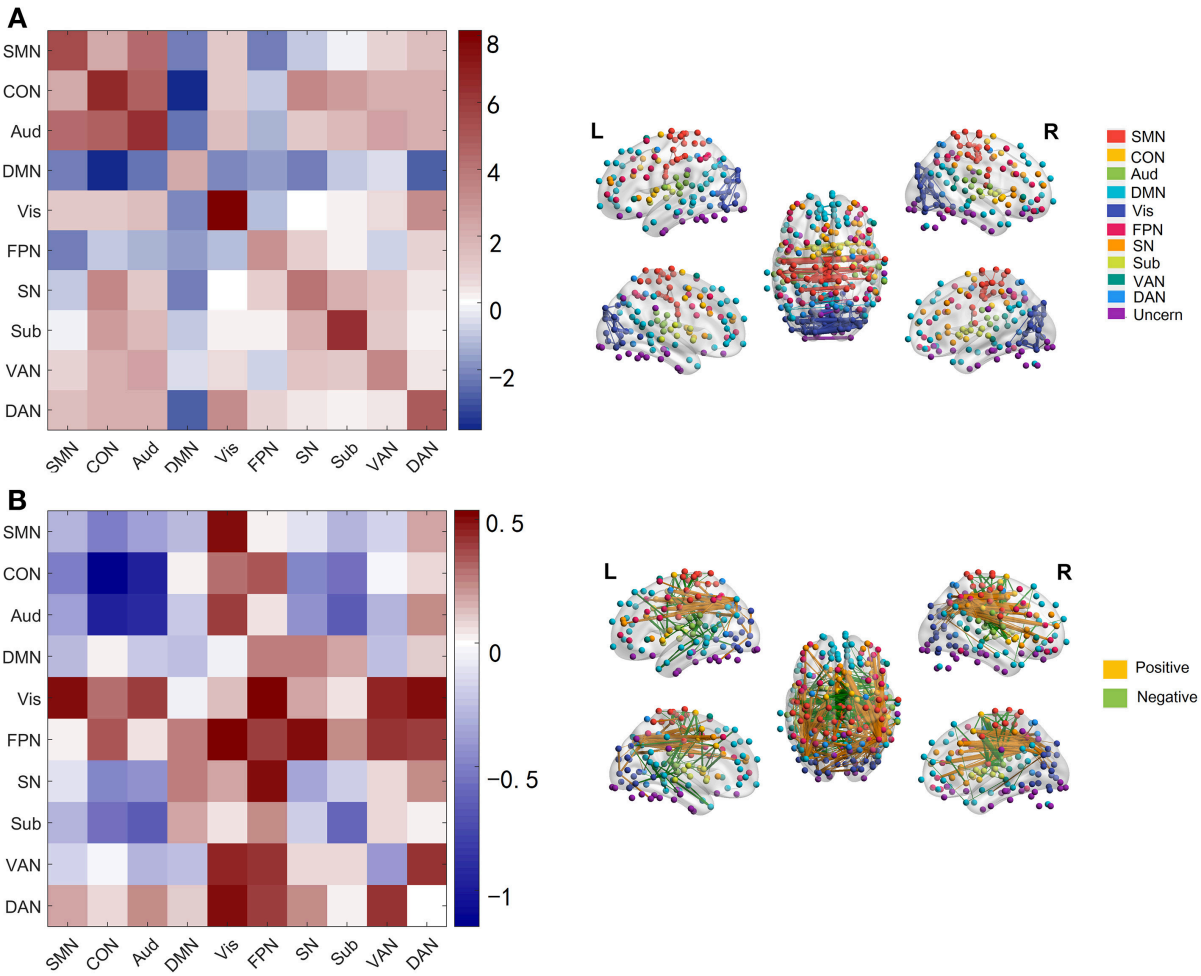
Networks and edges in PC 1 and PC 4. **(A)** Mean value of edges within and between networks in PC 1(left panel), and edges with absolute value higher than 12 in PC 1 (right panel) which are chosen for convenient viewing. **(B)** Mean value of edges within and between networks in PC 4 (left panel), and edges with absolute value higher than 1.8 in PC 4 (right panel).

We next investigated which connections and networks were prominent in PC 1 and 4 and the results were shown in Figure 3. Based on 10 networks identified in Power et al. (2011), we calculated the within-network value and between-network value of PC 1 and 4 as we did in Section Functional Connectivity Across the Lifespan. Meanwhile, the edges with higher absolute value were also extracted and plotted in Figure 3. As shown in Figure 3A and Supplementary Figure 4, the within-network connectivity or value was larger than between-network value for each network and between-network values of DMN with other networks such as CON and DAN were negative, which was highly consistent with functional networks identified by Power et al. (2011). As for PC 4, within-network values including CON, Aud, Sub and VAN, and between-network values including CON-Aud, Aud-Sub, SMN-CON, and CON-Sub were negative. Within-network value of FPN, and between-network values including SMN-Vis, Aud-Vis, Vis-FPN, Vis-VAN, Vis-DAN, FPN-SN, FPN-VAN, and VAN-DAN were positive (Figure 3B and Supplementary Figure 5). Relationship between values of age-effect matrix and values of PC 4 was fitted nicely by a linear model with a negative intercept (Figure 2B), which might explain why there were seemingly more positive values in PC 4 (Figure 3B).

We used another distinct brain template defined by Dosenbach et al. (2010) for a control analysis. The results of principal component analysis on the 160 × 160 FC matrices across all the subjects were quite similar (Supplementary Figures 1, 2). The first 100 principal components accounted for more than 70% of the total variance and coefficients of the fourth principal component across subjects were highly correlated with age vector (*r* = 0.59, *p* < 0.0001). The correlations between the first principal component and multi-subject matrix, and between the fourth principal component and age-effect matrix were as high as 0.99 (*p* < 0.0001) and 0.81 (*p* < 0.0001) respectively.

### Functional Connectivity Changes Across the Lifespan

**Figure 4:**
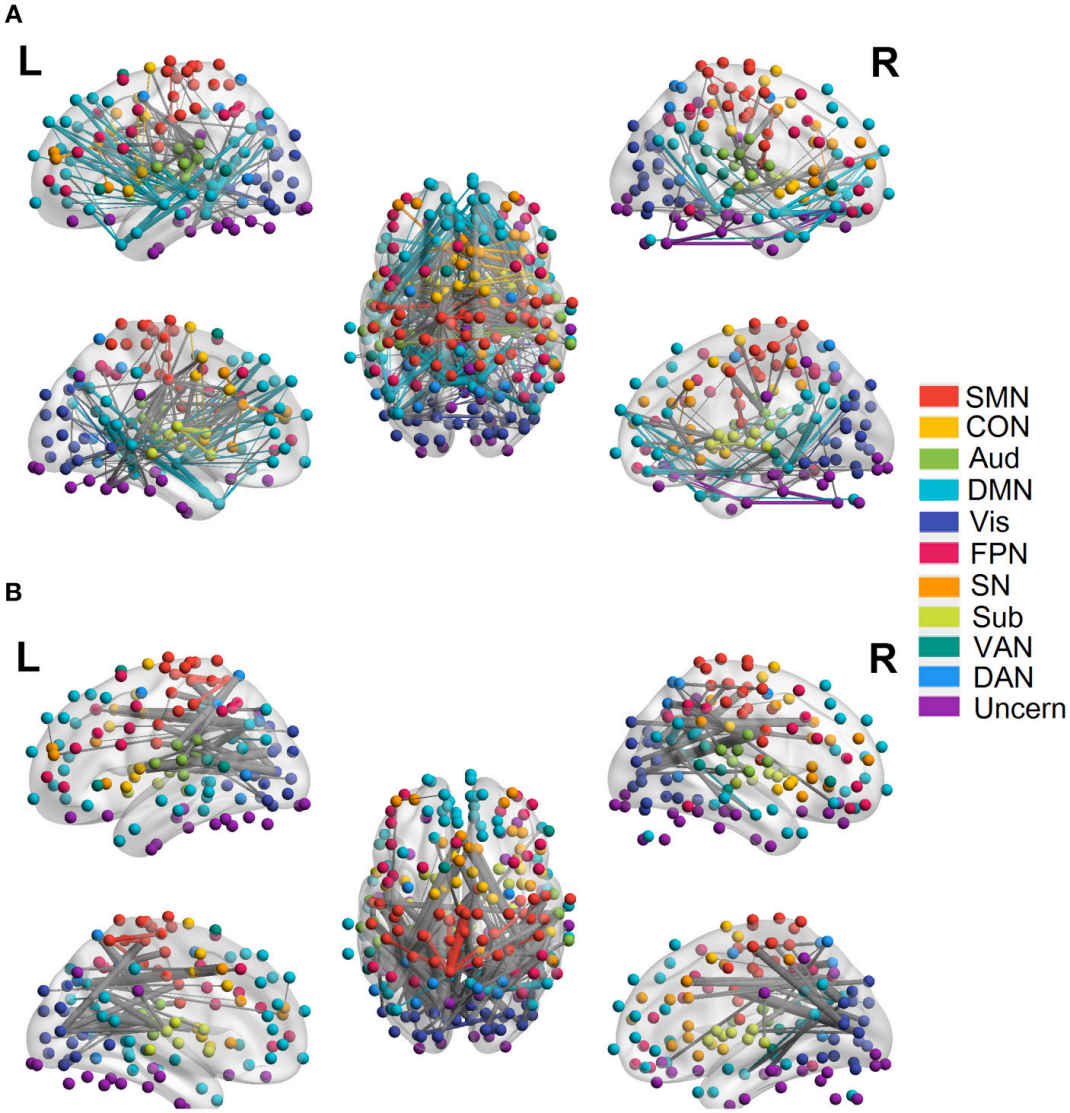
Linear relationship between functional connectivity and age at the edge level (*p* < 0.0001, FDR corrected). **(A)** Significant edges linearly decreasing with age. **(B)** Significant edges linearly increasing with age. Different color refers to different networks. If two nodes of the edge have same color, that is, they belong to the same network, the edge will be set as the same color. Otherwise the edge will be colored gray. The edge size is scaled by its T-statistics.

**Figure 5:**
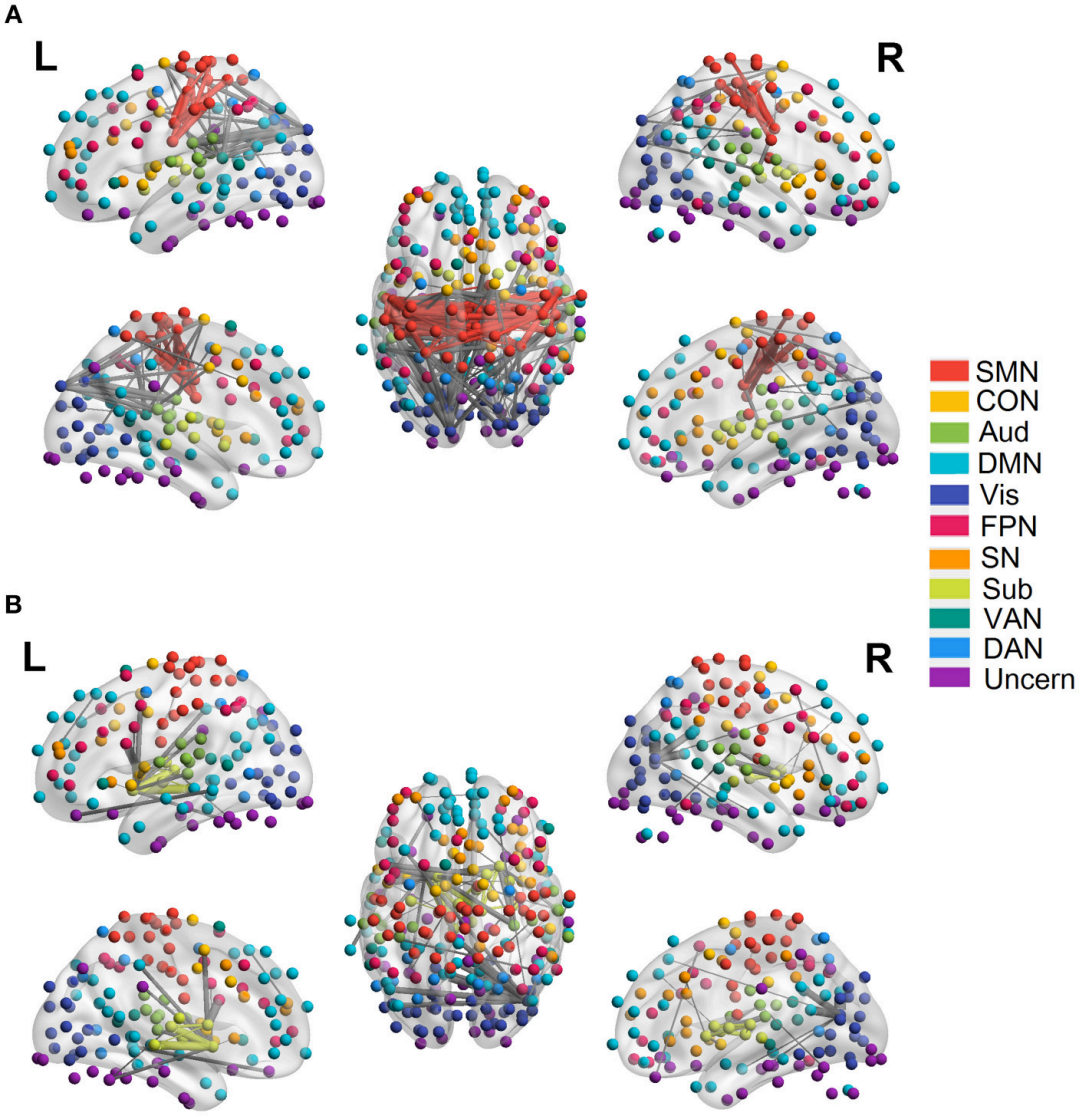
Quadratic relationship between functional connectivity and age at the edge level (*p* < 0.0001, FDR corrected). **(A)** Significant edges showing negative quadratic changes with age. **(B)** Significant edges showing positive quadratic changes with age. Different color refers to different networks. If two nodes of the edge have same color, that is, they belong to the same network, the edge will be set as the same color. Otherwise the edge will be colored gray. The edge size is scaled by its T-statistics.

We employed the functionally defined atlas and resting-state FC community in Power et al. (2011) and mainly focused on 10 subnetworks (Figure 4, color coded by different networks). At the edge-level, both linear and quadratic changes (*p* < 0.0001, FDR corrected) over age had been revealed across the functional connections (Figures 4, 5). Linear decreases with age were mainly found in within-network functional connections including CON, DMN and Aud, and between-network connections among DAN-Aud, SN-DMN, SMN-Vis, and DMN-Vis (Figure 4A). Linear increases with age were found in some within-network connections of SMN, and between-network connections including DMN-SMN, DMN-SN, DMN-Sub and SMN-SN, and connections between networks were more than within networks (Figure 4B). There were a small percentage of within-network connections in SMN, and between-network connections, including DMN-CON, SM-Aud, SM-Vis, and DMN-DAN, showing negative quadratic changes with age (Figure 5A). These connections showed early age-related increases and late age-related decreases (inverted U-shaped). Positive quadratic changes with age were mainly found in quite a small number of within-network connections in Sub as well as between-network connections among Vis-DMN, Sub-FPN, and Aud-Sub (Figure 5B). These connections showed early decreases and late increases (U-shaped) in life. Much more connections decreased with age linearly compared with the other three cases.

**Figure 6:**
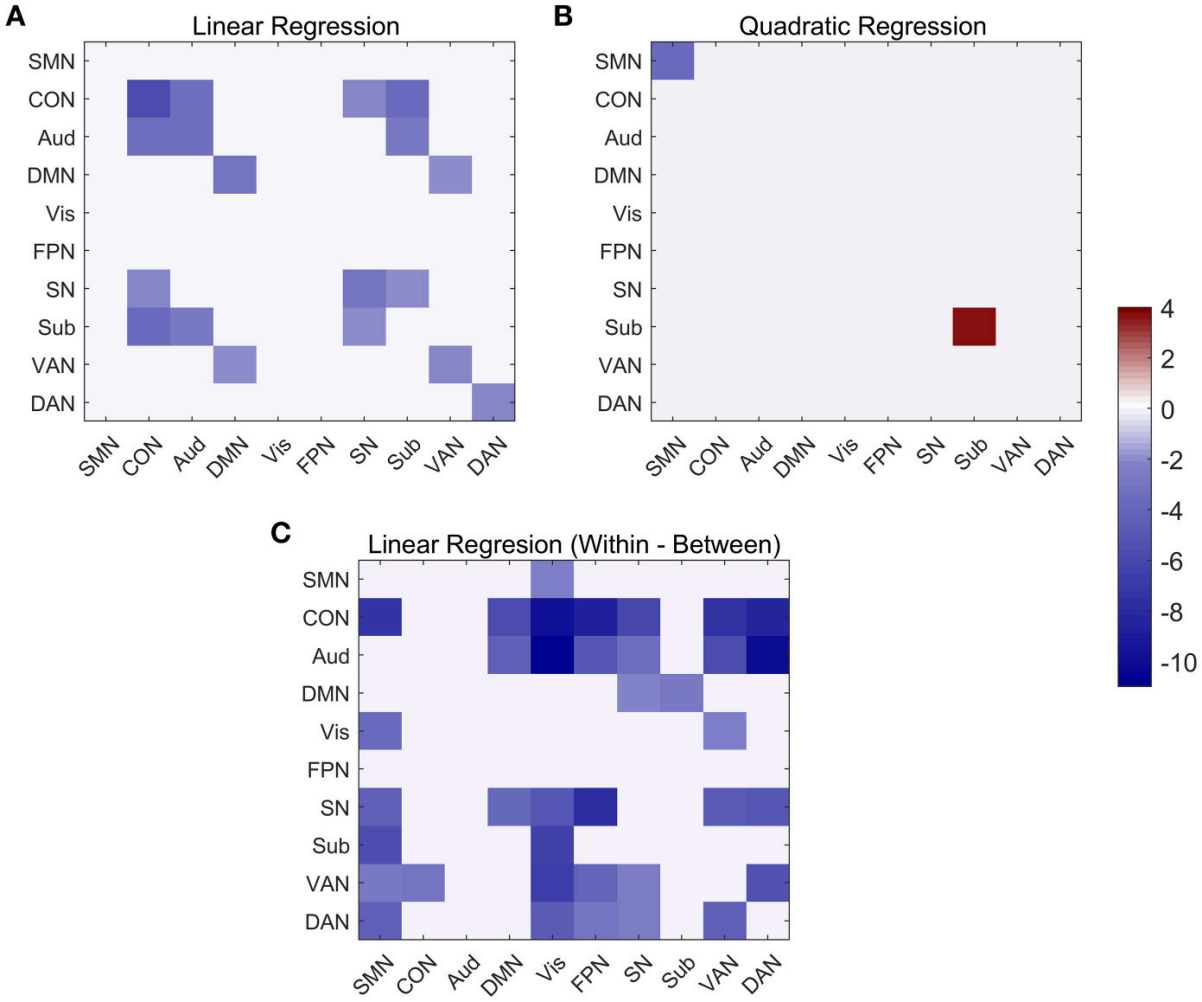
Linear and quadratic relationship between functional connectivity and age at the network-average level. **(A)** Significant networks showing linear changes with age in both within and between network connectivity. **(B)** Significant networks showing quadratic changes with age in both within-network and between-network connectivity. **(C)** Significant networks showing linear changes with age in within-between network connectivity.

**Figure 7:**
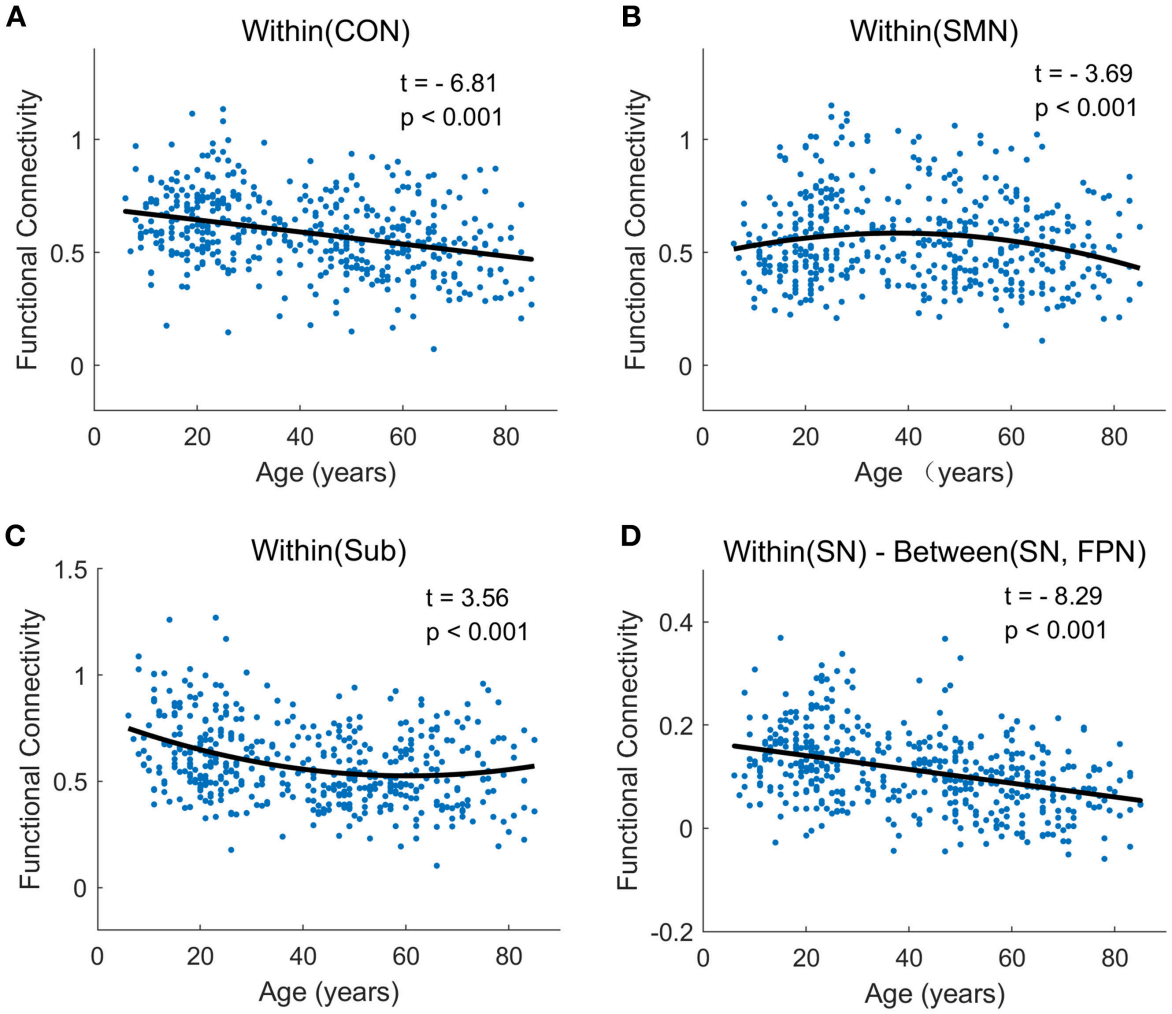
The typically developmental trajectories of functional connectivity at the network-average level. **(A)** Linear decrease with age in Within (CON) connectivity. **(B)** Negative quadratic change with age in Within (SMN) connectivity. **(C)** Positive quadratic change with age in Within (Sub) connectivity. **(D)** Linear decrease with age in Within (SN)-Between (SN, FPN) connectivity. The curve fits are shown by the dark lines.

At the network-level, linear and quadratic regression models were also employed to explore the associations of age with within-network, between-network and within-between network connectivity (Figure 6). Six networks, including CON, Aud, DMN, SN, VAN, and DAN, displayed linear decreases with age within network despite differing in the degree of age-related change (Figure 6A). Between-network connectivity, such as CON-Aud, CON-Sub, Aud-Sub, DMN-VAN, and SN-CON, also decreased with age (Figure 6A). Within-network of SMN demonstrated negative quadratic changes (inverted U-shaped) and Sub displayed positive quadratic changes (U-shaped) with age, respectively (Figure 6B). The above results were quite similar with findings in Figures 4, 5. While taking the difference between within-network and between-network connectivity for each network into consideration, it showed linear decrease with age in a number of networks, such as the difference between within-network of CON and between-network of CON and SMN which might be expressed by Within(CON)-Between(CON, SMN) (Figure 6C). Examples of the six types of changes with age were exhibited in Figure 7.

### Temporal Measures Across the Lifespan

For each subject, we computed and acquired their fALFF maps, after which we calculated the correlations between age and fALFF values for each voxel. The resultant correlation maps for fALFF are depicted in Supplementary Figure 3. The *p*-values for fALFF correlation maps were FDR corrected for multiple comparisons (FDR *p* < 0.05). Apart from some regions in SN, DMN, Sub and Aud, the majority of the brain showed statistically significant negative correlations between fALFF and age. Complementarily, we explored the linear or quadratic relationships between age and average values of fALFF for each node within the template we employed. For fALFF map, two types of typical changes with age were found (Supplementary Figure 3B). Positive quadratic changes with age were found in some nodes of DMN, DAN, Sub, CON and SMN, though the majority of nodes showed linear decreases with age (FDR *p*-value < 0.001).

### Prediction Results

In the present work, three feature selection/reduction methods and three regression models, were constructed based on the datasets. The models were tested in the dataset NKI-RS-E using *K*-fold cross-validation method. Models trained in the entire NKI-RS-E data set were further validated externally in NKI-RS dataset. The predictive performance was assessed by two statistical criterions Pearson correlation coefficient and MAE between the predicted ages and chronological ages across subjects.

**Figure 8:**
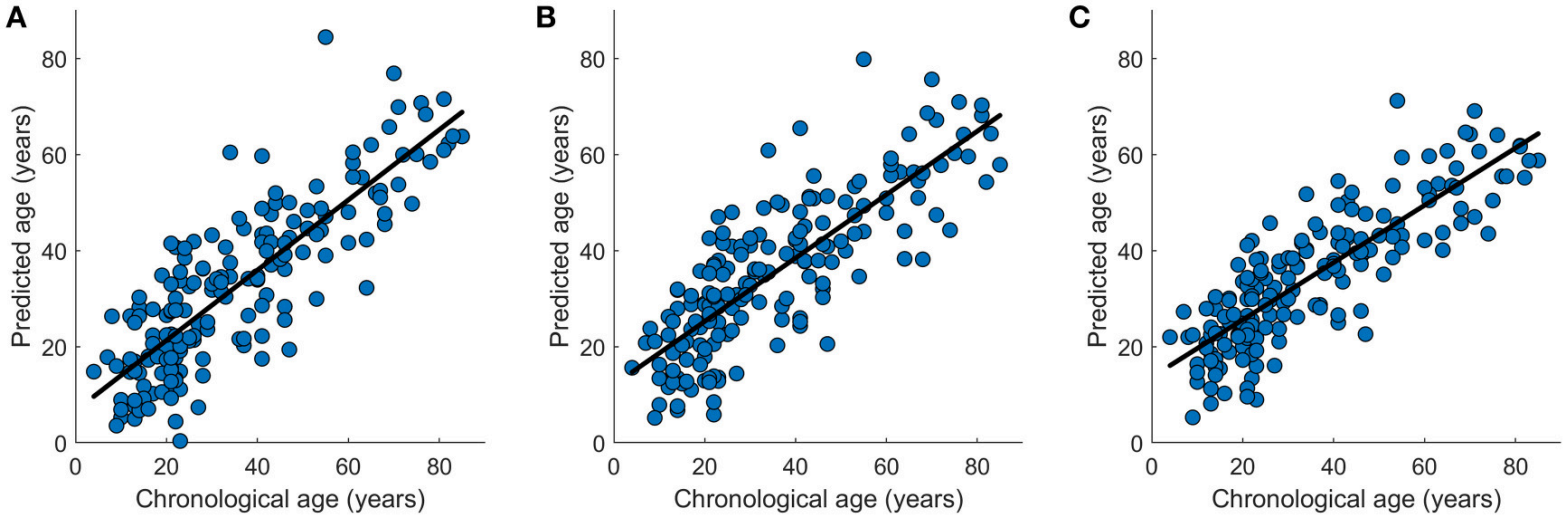
Graphical representation of the age prediction results. **(A)** Chronological age (x-axis) vs. predicted age (y-axis) acquired by OLS regression model. **(B)** Chronological age (x-axis) vs. predicted age (y-axis) acquired by SVR regression model. **(C)** Chronological age (x-axis) vs. predicted age (y-axis) acquired by Lasso regression model. The curve fits are shown by the dark lines.

In the data set NKI-RS-E, *K*-fold cross-validation was performed and the accuracies for all *K*-fold rounds were averaged together to generate the internal accuracy of prediction. Twelve models were trained as displayed in Tables 1, 2. In the network-based feature selection section, the number of PCs was set to 150 for higher number than 150 did not change the performance. In the Lasso regression training process, we went through parameter λ to get the best penalization parameter. As for the SVR, different kernel functions were employed and the one which had the best prediction performance was picked each time the SVR predictive model was trained. The best prediction results for 12 predictive models are displayed in Tables 1, 2. The brain age of subjects in the datasets was accurately estimated using all the predictive models. Lasso regression model combined with network-based feature extraction method provided a better prediction result: Pearson correlation coefficient *r* = 0.910 (*p* < 0.0001) and MAE = 6.5 years for internal validation, *r* = 0.838 (*p* < 0.0001) and MAE = 8.8 years for external validation. Compared with network-based method and edge-based method, the temporal feature method did not show a comparative prediction performance (Steiger test, *p* < 0.05). The chronological age vs. predicted age acquired by three regression models combined with network-based feature extraction method were plotted in Figure 8.

**Table 1:** Comparison of prediction performance using different predictive models.

**Feature method**		**Network-based**		**Edge-based**	

**Regression model**		**NKI-RS-E (*K*-fold)**	**NKI-RS (external)**	**NKI-RS-E (*K*-fold)**	**NKI-RS (external)**
OLS	Pearson's correlation	0.846 (*p* < 0.0001)	0.786 (*p* < 0.0001)	0.867 (*p* < 0.0001)	0.828 (*p* < 0.0001)
	MAE (years)	7.9	9.8	6.7	9.3
SVR	Pearson's correlation	0.868 (*p* < 0.0001)	0.769 (*p* < 0.0001)	0.890 (*p* < 0.0001)	0.832 (*p* < 0.0001)
	MAE (years)	7.5	9.9	7.1	9.4
LASSO	Pearson's correlation	0.910 (*p* < 0.0001)	0.838 (*p* < 0.0001)	0.896 (*p* < 0.0001)	0.835 (*p* < 0.0001)
	MAE (years)	6.5	8.8	6.9	9.2

*Feature selection methods: network-based method and edge-based method; regression model: ordinary linear regression (OLS method), SVR and Lasso*.

**Table 2:** Prediction performances of models combined with temporal feature extraction methods (fALFF) and regression models.

	**fALFF**	
**Regression model**	**Pearson's correlation**	**MAE (years)**
OLS	0.668 (*p* < 0.0001)	14.2
SVR	0.753 (*p* < 0.0001)	11.4
LASSO	0.817 (*p* < 0.0001)	9.2

Based on the Lasso regression model combined with network-based feature extraction method, we performed z-test on the external predictive results, which showed no significant gender-related differences (*z* = 1.45 < 1.96). Then we correlated the MAE values with age across subjects, resulting in a correlation of 0.469 (*t* = 6.77, *p* < 0.001) and suggesting significant age-related prediction differences.

### Control Analysis for Predictive Model

We used another distinct template (Dosenbach et al., 2010) to test whether the choice of templates had an effect on the prediction performance. Different feature selection/reduction methods were conducted, and the three regression models were learned: ordinary linear regression (OLS regression), SVR, and Lasso. The prediction models displayed a comparable prediction performance with the Power-264 template (average correlation *r* > 0.8) and the results are shown in Supplementary Tables 3, 4. Similarly, Lasso regression model combined with network-based feature extraction method showed a better accuracy: *r* = 0.895 (*p* < 0.0001) and MAE = 6.8 years for internal validation, *r* = 0.821 (*p* < 0.0001) and MAE = 9.3 years for external validation. Meanwhile, we also employed the method proposed in Dosenbach et al. (2010) to our datasets and the prediction performance was presented in Supplementary Table 5: *r* = 0.724 (*p* < 0.0001) and MAE = 12.1 years for internal validation, *r* = 0.621 (*p* < 0.0001) and MAE = 17.7 years for external validation. Then we compared its performance with the prediction result of network-based Lasso model using Steiger's *z*-test, which indicated the later was significantly more accurate (Steiger's *z* = 7.35, *p* < 0.001).

### Significant Edges and Networks in Predictive Model

**Figure 9:**
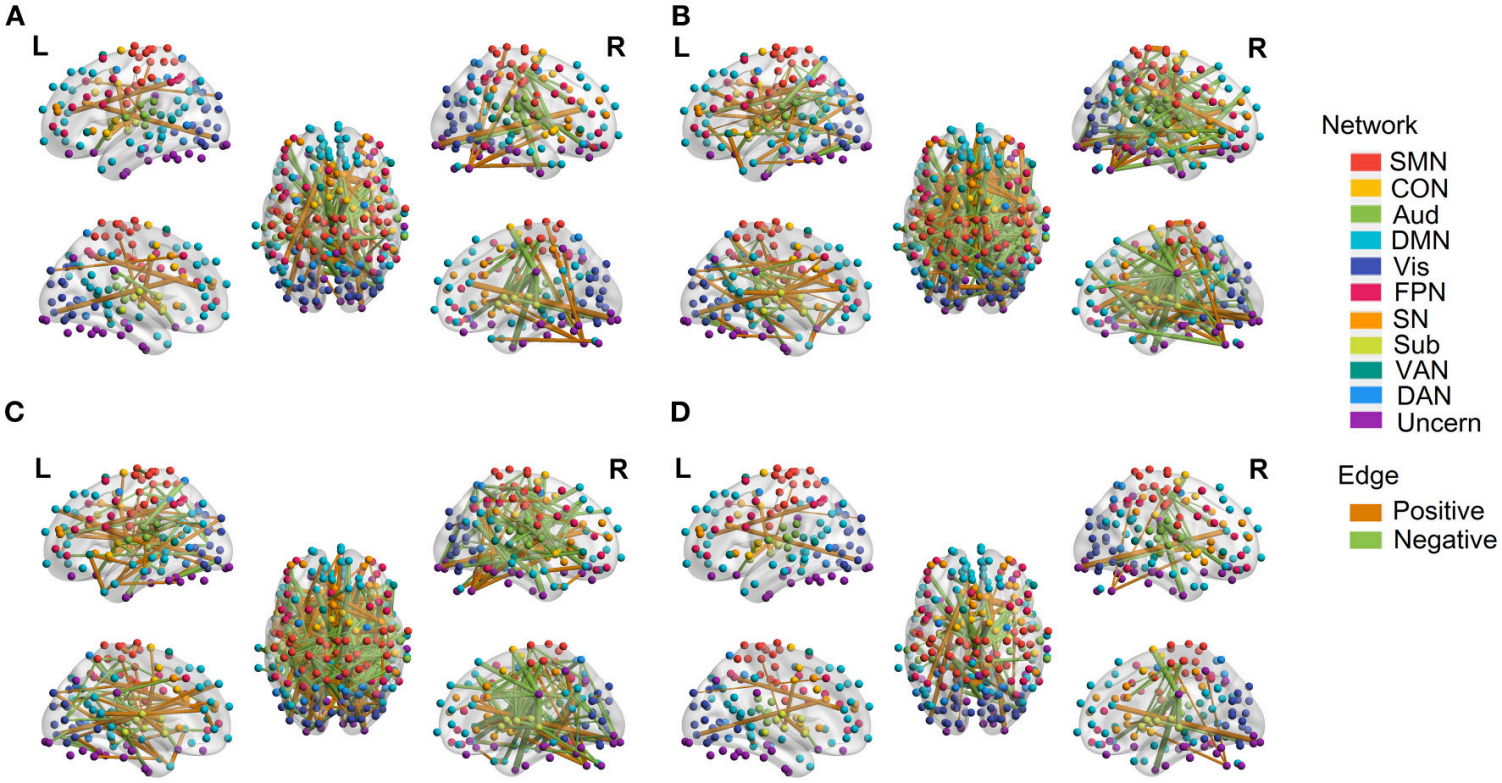
Edges with significant weights in predictive models combining three regression models with edge-based feature selection method. **(A)** Edges with significant weights in OLS regression model. **(B)** Edges with significant weights in SVR regression model. **(C)** Edges with significant weights in Lasso regression model. **(D)** Common edges with significant weights in all three models. Edges with positive significant weights are shown in orange, whereas edges with negative weights are shown in green.

**Figure 10:**
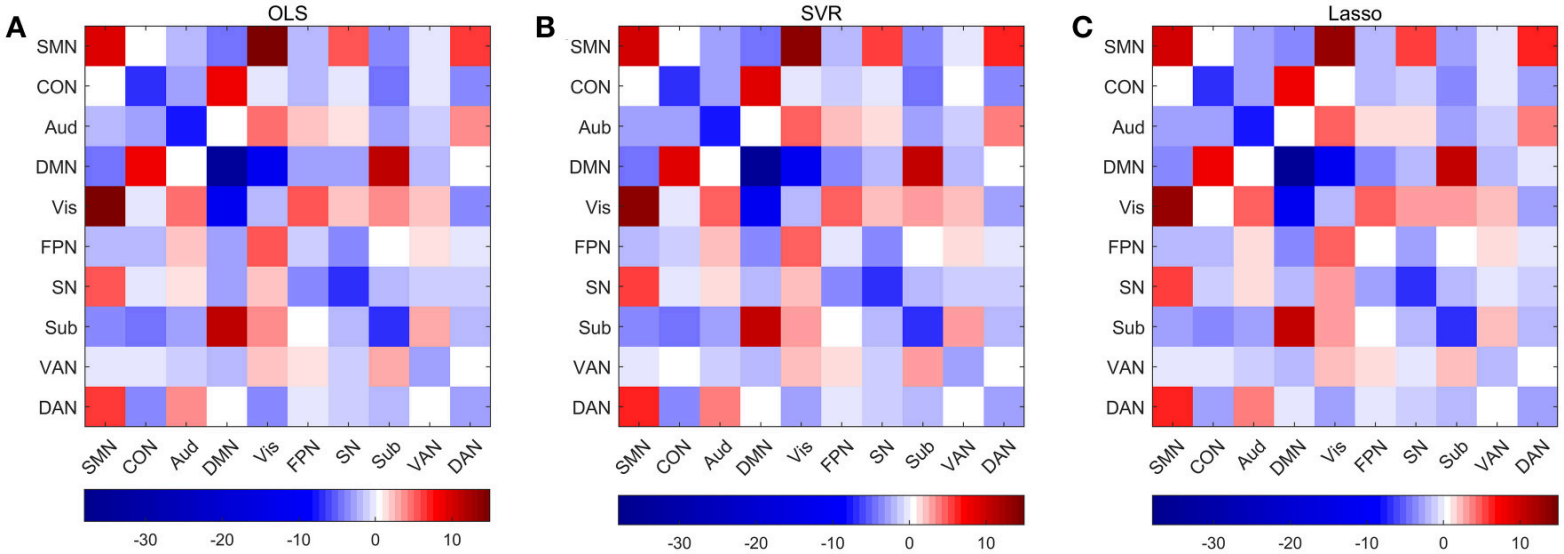
Average weights of edges within and between networks in predictive models combining three regression models with edge-based feature selection method. **(A)** Average weights within and between networks in OLS model. **(B)** Average weights within and between networks in SVR model. **(C)** Average weights within and between networks in Lasso model.

Permutations were performed to explore edges with statistically significant weights in the three regression models combined with edge-based feature selection/reduction method (two-tailed *p* < 0.001, Figure 9 and Supplementary Figures 6–9 with greater clarity). We found that 41 common edges with significant positive weights, 24 common edges with significant negative weights in three regression models to predict brain age and these significant edges were distributed across the brain. Then we estimated the relative contributions of particular networks to age prediction by summing up the weights of edges in three predictive models within network and between networks (Figure 10). At the network level, three regression models, OLS, SVR, and Lasso, displayed quite similar results. Edges within CON, Aud, DMN, SN and Sub, and between-network, including DMN-Vis, exhibited negative weights (trending to predict younger ages). Within-network connectivity, including SMN, and between-network connectivity, including SMN-Vis, SMN-DAN, DMN-CON, and DMN-Sub, displayed positive weights (trending to predict older ages).

## Discussion

Cognitive functioning performance is closely related to age and much work has been done trying to disclose how aging affects integration of information within and between functional networks (Song et al., 2014; Geerligs et al., 2015a; Bassett and Sporns, 2017; Damoiseaux, 2017; Grayson and Fair, 2017). In the present study two independent datasets were employed and functional connectivity network was constructed based on the preprocessed fMRI data. Network-based principal component analysis on the FC matrices across all the subjects and we probed the acquired components in a detail way. Apart from coefficients across PCs, edges of functional connectivity and temporal measures, fALFF, were also included as features, and linear and quadratic regression models were applied to explore the correlations with age of these features. Based on different types of features, feature selection/reduction methods were proposed and three regression models were adopted. The predictive models were trained on the datasets and displayed good prediction performance.

In the network-based principal component analysis, the PC 1 was not only similar with individual's FC matrix (mean correlation *r* = 0.59, *p* < 0.0001), but highly correlated with the multi-subject matrix (correlation *r* = 0.98, *p* < 0.0001) which was defined by calculating correlations across the concatenated time series within nodes of all subjects. Thus, we could regard PC 1 as the intrinsic component. We also observed that the principal component coefficients of the PC 4 had a correlation coefficient as high as 0.69 (*p* < 0.0001) to age vector. What's more, the fourth principal component was highly similar (*r* = 0.88, *p* < 0.0001) with the age-effect matrix which was acquired by computing the correlation between age and functional connectivity for each ROI-pair, suggesting that PC 4 could be treated as the age-related component. Therefore, the resting state functional architecture for one subject is mainly shaped by intrinsic component, age-related component and other components. The first principal component of the analysis accounted for approximately 36% of the total variance in inter-subject network architecture, while the fourth component accounted for less than 2% of the total variance, which indicates that the age-related changes are relatively small but functionally important. To further probe into PC 4, a simple linear regression model was applied to explore the relationship between edge value in PC 4 and resting state FC matrix (Figure 2B) and another linear regression model to probe the relationship between age and coefficient of PC 4 across subjects (Figure 1D). For the loading coefficients of PC 4, coefficient of PC 4 at age around 40 was about 0, children, adolescents and young people under age 40 have negative coefficient, while older people tend to have positive coefficient of PC 4. Thus the PC 4 might be considered as the component which accounts for the majority of the linear changes between resting-state brain network of subjects aged around 40 and other age groups. As for the edge value in PC 4, the larger the absolute values of edge in PC 4, the more influence age will exert on the corresponding edge in resting state FC network (either positive or negative, while negative dominant). What's more, Chi-square test was performed to test whether there might be significant differences between the number of inter-hemispheric and the number of intra-hemispheric edges. Compared with inter-hemispheric edges, there seem to be significantly more intra-hemisphere edges in PC 4. Especially decreased intra-hemisphere edges were found within functional networks like SMN, DMN, and Aud, which is consistent with linear reduced within-network functional connectivity in these networks Iin our findings of edge-based method.

At the edge-level, a number of edges within DMN displayed linear decrease with age in functional connectivity, which is consistent with previous findings (Mevel et al., 2011; Tomasi and Volkow, 2011; Hafkemeijer et al., 2012; Douaud et al., 2014; Geerligs et al., 2015a). DMN might be the most investigated resting state network and is linked to a variety of cognitive processes such as episodic memory, self-referential processing, and mind wandering (Christoff et al., 2009; Andrews-Hanna, 2012; Raichle, 2015). Thus, age-related decreases in functional connectivity within DMN might explain worse performance on cognitive tasks like memory of older people. Linear reduced functional connectivity in edges were also found within CON and this is consistent with previous research (Geerligs et al., 2015a). Among edges which showed linear increases with age, more were found in between-network connections than within-network, especially between networks of Vis, SMN and Aud, and this is consistent with the finding that participation coefficients of visual and Somato-motor networks were increased in older adults in Geerligs et al. (2015a). A variety of edges within SMN showed negative quadratic changes with age, which is also strengthened by finding at the network-level (Figure 5B). Edges of this kind exhibited increases in functional connectivity in adolescents and younger adults, whereas decreases in elderly. Studies have revealed age-related differences in sensorimotor cortex (Wu et al., 2007; Heuninckx et al., 2008) and indicated compensatory mechanism in aging brain as not only classical motor coordination regions, but also higher-level sensorimotor regions, and frontal regions are activated in the older adults (Heuninckx et al., 2008). Chan et al. (2014) found that age function for sensory-motor network was fit significantly only by a linear model, which is inconsistent with our finding of negative quadratic changes with age in SMN. The difference in datasets as well as methodology might explain this discrepancy, subjects aged under 20 were not included and nuisance regressors such as head motion and brain volume were not regressed out in the regression model in Chan et al. (2014).

At the network-average level, six networks out of ten, including CON, Aud, DMN, SN, VAN and DAN, exhibited linear reduced within-network functional connectivity. This is consistent with previous findings (Chan et al., 2014; Spreng et al., 2016). Spreng et al. (2016) found reduced within-network functional connectivity across DMN and DAN networks in older adults compared to younger people. Chan et al. (2014) employed networks identified in Power et al. (2011) likewise, calculated the within-network functional connectivity of ten networks and found linear decreases with age. Between-network functional connectivity was also found to increase with age in Chan et al. (2014), Damoiseaux (2017), however, that is not the case in our research. Although edges with increased functional connectivity between networks were found at the edge-level (Figure 4), we did not find significantly increased between-network connectivity at the network-level. This discrepancy might arise in part from differences in methodology, for instance, gender, total intracranial volume and head motion had been considered as nuisance regressors and regressed out in our case. To make it clear how between-network connectivity correlated with age, we further calculated the difference between within-network connectivity for one network and between-network connectivity which connected it with all other nine networks, and applied linear regression model to explore the associations with age. As we expected, linear decreases with age in within-between network connectivity had been discovered among almost all networks. This means within-network connectivity decreases with age more rapidly than between-network connectivity and leads to decreasing network segregation, a measure of between-network connectivity relative to within-network connectivity defined in Chan et al. (2014). Thus the elderly seem to have lower brain network segregation and specialization, which is consistent with earlier findings (Tomasi and Volkow, 2011; Chan et al., 2014; Grady et al., 2016; Damoiseaux, 2017).

We performed permutations to find edges with significant weights in three predictive models with edge-based feature reduction method. Sixty-Five common edges with significant weights were found in all three models, while 41 edges with positive weights and 24 edges with negative weights. We calculated the predictive power of nodes by summing up the weights of all the connections to and from that node and found that one node in the region of Cingulate Gyrus had the highest relative predictive power. We then compared the 65 common edges with significant weights in predictive models with edges showing significant linear or quadratic changes with age in regression models. Beyond our expectation, only 24 out of 65 edges displayed significant developmental trajectories across the lifespan, which signifies that edges with significant weights to predictions of age do not necessarily display significant linear or quadratic changes over age at the individual-edge level and vice versa. One explanation might be multicollinearity is so prevalent in the tremendous number of edges and some edges are redundant with respect to others. What's more, high-dimensional functional connectivity space was transformed to a feature space with lower dimension through the edge-based feature reduction method which takes not only correlation with age but also variance maximization into consideration simultaneously, thus edges would be assigned weights in predictive model more subtly. We also summed the weights of edges in all three models both within and between networks, showing quite similar results and indicating that edge-based feature reduction process exerts more influence on the predictive performance than subsequent regression models like OLS or SVR. In Dosenbach et al. (2010) it was found that majority of within-network connections had positive weights, whereas between-network displayed negative weights, which is quite different from results in present research (Figure 9). There might be two reasons for this disparity. On one hand, dataset only included subjects aged 7 to 30 years in Dosenbach et al. (2010) while it is more difficult to learn the complicated development model of brain. On the other hand, there are considerable differences in methodology especially the feature selection methods.

Models trained on temporal features (fALFF) did not have expected and comparable prediction performances with the other two feature selection/reduction methods (Steiger test, *p* < 0.05). One reason that might lead to this is that fALFF do not contain enough information related with age as functional connectivity does. Moreover, fALFF identifies the proportion of the observed signal in the low frequencies (0.01–0.1 Hz) compared to the entire range of BOLD frequencies, suggesting that it is specific to low frequencies. However, studies have shown that multiple frequency bands of BOLD signals provide meaningful information (Chen and Glover, 2015; Gohel and Biswal, 2015; Yue et al., 2015). Age-related changes across multiple frequency bands need to be considered in the future.

The predictive performance in external validation seems not to be as good as in the internal validation. There might be three reasons which lead to the divergence between predicted ages and chronological ages. Firstly, variations between internal dataset and external dataset could introduce additional prediction error. The internal dataset, i.e., NKI-RS-E dataset, provides data sets with faster repetition time (0.645 s) and higher resolution than external NKI-RS dataset (TR = 2.5 s) (Nooner et al., 2012). The time for resting state fMRI scan is 10 min for both datasets, thus shorter TR means larger number of volume of RS-fMRI images (900 vs. 260 volumes). In our future study, dataset with higher-quality data, such as provided by the Human Connectome Project, would be included to assess the error introduced by the inter-dataset variance. Secondly, the predicted brain age for a subject may differ from his/her chronological age because of inter-individual variations in experience, health and gene, which can lead to a systemic error. For example, people with age-associated brain disease might deviate from healthy brain-aging trajectories. Thirdly, there might exist some complex age-related changes with age that all the learning models in our study have not accounted for. Although performance of external validation is not as good as the internal validation, our research does suggest that sufficient information could be extracted from RS-fMRI data to make prediction about individual brains' functional development level.

There are also some limitations which need to be considered in the future study. Firstly, all the data included here was acquired under the resting state for RS-fMRI data was the easier to collect and aggregate across subject populations and sites. However, functional connectivity also depends on the individual's mental state (Cole et al., 2014; Geerligs et al., 2015b; Dubois, 2016; Bassett and Sporns, 2017). Thus, future work should take task-based fMRI data into consideration to test how the age-state interaction affects individual's functional architecture and whether different mental states have an effect on the performance of the predictive models. Secondly, a variety of studies have analyzed the complex dynamic characteristics of functional connectivity, challenging the assumption that functional connectivity between brain regions is static during the duration of the resting time (Hutchison and Morton, 2015; Davison et al., 2016; Battaglia et al., 2017; Preti et al., 2017; Tian et al., 2018). We will test whether the principal component analysis results and prediction performance still hold while using dynamic functional connectome methods. Finally, even though age range of all the subjects included in the present work was across the lifespan, the fMRI data was usually acquired within 1 day and there exists few longitudinal studies investigating lifespan changes of functional connectivity. Recent studies have shown that human brain structure and function not only vary across individuals but also across time at different time scales (Bassett and Sporns, 2017; Peña-gómez et al., 2017; Preti et al., 2017; Shine and Poldrack, 2017). We still do not have a fully understanding of how the brain undergo changes over months, years or even across the lifespan for an individual. Future work should pay more attention to the longitudinal research.

In conclusion, we performed principal component analysis to a large sample size of dataset and acquired meaningful components. Then three different feature selection/reduction methods were proposed and regression models, OLS, SVR, and Lasso were trained based on the feature extraction results. The learned predictive models provided a comparatively accurate prediction of age for individuals, meaning sufficient information could be extracted from resting state fMRI data to model brain development across lifespan. Based on our prediction results of different regression models combined with distinct feature selection/reduction methods, edge-based feature selection/reduction method might be a better choice in feature extraction stage as learned models based on it exhibited more robust prediction results. As for SVR and Lasso, models based on Lasso do not reveal significant advantages over SVR, but Lasso might be a preferred regression model for it not only provides a comparable prediction performance if not better but also is more time-efficient.

## Author Contributions

All authors listed have made a substantial, direct and intellectual contribution to the work, and approved it for publication.

### Conflict of Interest Statement

The authors declare that the research was conducted in the absence of any commercial or financial relationships that could be construed as a potential conflict of interest.
