# Supplementary material for: Predicting Brain Age Based on Spatial and Temporal Features of Human Brain Functional Networks
Source: Front Hum Neurosci. 2019 Feb 26;13:62. doi: 10.3389/fnhum.2019.00062 (PMC6399206; doi:10.3389/fnhum.2019.00062)
Supplement: Supplementary file 1 [file Table_1.DOCX]

Supplementary Material

# Supplementary Figures and Tables

## Supplementary Figures

**Supplementary Figure 1.** Principal component analysis results through employing another distinct template (Dosenbach et al., 2010). (A) Variance explained by the first ten components. (B) Variance explained by the first 100 components. (C) The first ten principal components which were most closely correlated with age. (D) The coefficients of PC 4 across subjects (y-axis) versus ages of subjects (x-axis) fitted by a linear regression model.

**Supplementary Figure 2.** Further analysis of principal component 1 and principal component 4 while using template in (Dosenbach et al., 2010). (A) PC 1 (left panel), multi-subject matrix by calculating correlations across the concatenated time series of all subjects (middle panel) and the quadratic association between values of PC 1 (x-axis) and values of multi-subject matrix (y-axis) (right panel). PC 1 and multi-subject matrix are highly correlated (r=0.99). (B) PC 4 (left panel), the matrix of age effects on edges by computing the correlation between age and functional connectivity for each edge (middle panel) and the linear association between values of PC 4 (x-axis) and values of age-effect matrix (y-axis) (right panel). PC 4 and age-effect matrix are highly correlated (r=0.81).

**Supplementary Figure 3.** Temporal changes with age in fALFF. (A) T-maps for fALFF when correlated with age across the subjects (p<0.05, FDR corrected). (B) Linear and quadratic relationship between fALFF values within nodes with age (p<0.001, FDR corrected).

**Supplementary Figure 4**. A figure with greater clarity for the right panel of Figure 3A: edges with absolute value higher than 12 in PC 1 which are chosen for convenient viewing.

**Supplementary Figure 5**. A figure with greater clarity for the right panel of Figure 3B: edges with absolute value higher than 1.8 in PC 4.

**Supplementary Figure 6**. A figure with greater clarity for Figure 9A: edges with significant weights in OLS regression model.

**Supplementary Figure 7**. A figure with greater clarity for Figure 9B: edges with significant weights in SVR regression model.

**Supplementary Figure 8**. A figure with greater clarity for Figure 9C: edges with significant weights in Lasso regression model.

**Supplementary Figure 9**. A figure with greater clarity for Figure 9D: common edges with significant weights in all three models.

## Supplementary Tables

**Supplementary Table 1.** Characteristics of subjects in NKI-RS-E dataset.

**Supplementary Table 2.** Characteristics of subjects in NKI-RS dataset.

**Supplementary Table 3.** Prediction performances using another distinct brain template (Dosenbach et al., 2010) for control analysis. Feature selection methods: network-based method and edge-based method; regression model: ordinary linear regression (OLS method), SVR and Lasso.

**Supplementary Table 4.** Prediction performances of models combined with temporal feature extraction methods (fALFF and ALFF) and regression models using different template (Dosenbach et al., 2010).

**Supplementary Table 5.** Prediction performances by adopting model construction method in (Dosenbach et al., 2010).

| Age | Number | Gender (M/F) | Handedness (R/L) |
| --- | --- | --- | --- |
| $\leq10$ | 13 | 9/4 | 10/2 (1 ambidexterous) |
| 11-20 | 82 | 43/39 | 74/7 (1 ambidexterous) |
| 21-30 | 96 | 53/43 | 80/11 (5 ambidexterous) |
| 31-40 | 39 | 13/26 | 36/1 (2 ambidexterous) |
| 41-50 | 75 | 16/59 | 69/6 |
| 51-60 | 68 | 12/56 | 59/9 |
| 61-70 | 58 | 20/38 | 50/6 (2 ambidexterous) |
| 71-80 | 35 | 13/22 | 32/3 |
| $\geq81$ | 9 | 2/7 | 9/0 |

| Age | Number | Gender (M/F) | Handedness (R/L) |
| --- | --- | --- | --- |
| $\leq10$ | 8 | 4/4 | 6/2 |
| 11-20 | 34 | 20/14 | 30/3 (1 ambidexterous) |
| 21-30 | 46 | 27/19 | 39/5 (1 unknown, 1 ambidexterous) |
| 31-40 | 20 | 11/9 | 16/4 |
| 41-50 | 26 | 21/5 | 23/3 |
| 51-60 | 12 | 8/4 | 10/2 |
| 61-70 | 14 | 7/7 | 12/2 |
| 71-80 | 8 | 3/5 | 7/1 |
| $\geq81$ | 5 | 3/2 | 5/0 |

| Feature method  Predictive model | | Network-based | | Edge-based | |
| --- | --- | --- | --- | --- | --- |
|  |  | NKI-RS-E (K-fold) | NKI-RS (external) | NKI-RS-E (K-fold) | NKI-RS (external) |
| OLS | Pearson’s correlation | 0.831 (p<0.0001) | 0.820 (p<0.0001) | 0.892 (p<0.0001) | 0.829 (p<0.0001) |
|  | MAE | 9.2 | 9.7 | 7.5 | 9.1 |
| SVR | Pearson’s correlation | 0.824 (p<0.0001) | 0.796 (p<0.0001) | 0.873 (p<0.0001) | 0.814 (p<0.0001) |
|  | MAE (years) | 9.4 | 10.8 | 7.8 | 9.3 |
| LASSO | Pearson’s correlation | 0.895 (p<0.0001) | 0.821 (p<0.0001) | 0.868 (p<0.0001) | 0.834 (p<0.0001) |
|  | MAE (years) | 6.8 | 9.3 | 7.6 | 9.0 |

|  | fALFF | |
| --- | --- | --- |
| Regression model | Pearson’s correlation | MAE (years) |
| OLS | 0.766 (p<0.0001) | 10.9 |
| SVR | 0.787 (p<0.0001) | 10.7 |
| LASSO | 0.806 (p<0.0001) | 9.6 |

|  | | NKI-RS-E (K-fold) | NKI-RS (external) |
| --- | --- | --- | --- |
| Dosenbanch’s method | Pearson’s correlation | 0.724 | 0.621 |
|  | MAE (years) | 12.1 | 17.7 |
