# Supplementary figures and images for: Predicting Brain Age Based on Spatial and Temporal Features of Human Brain Functional Networks
Source: Front Hum Neurosci. 2019 Feb 26;13:62. doi: 10.3389/fnhum.2019.00062 (PMC6399206; doi:10.3389/fnhum.2019.00062)

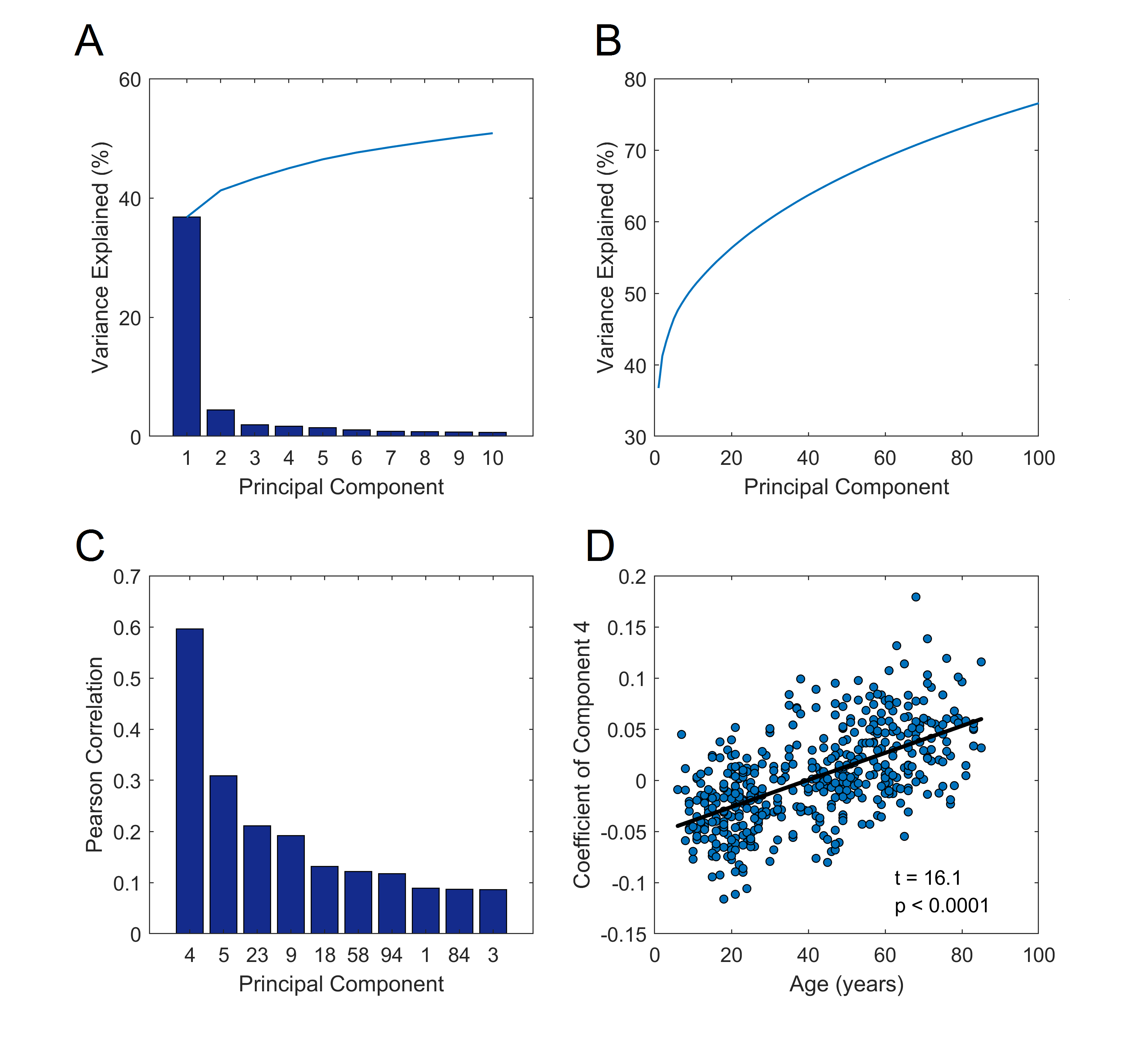

Supplement: Supplementary file 2 [file Image_1.TIF]

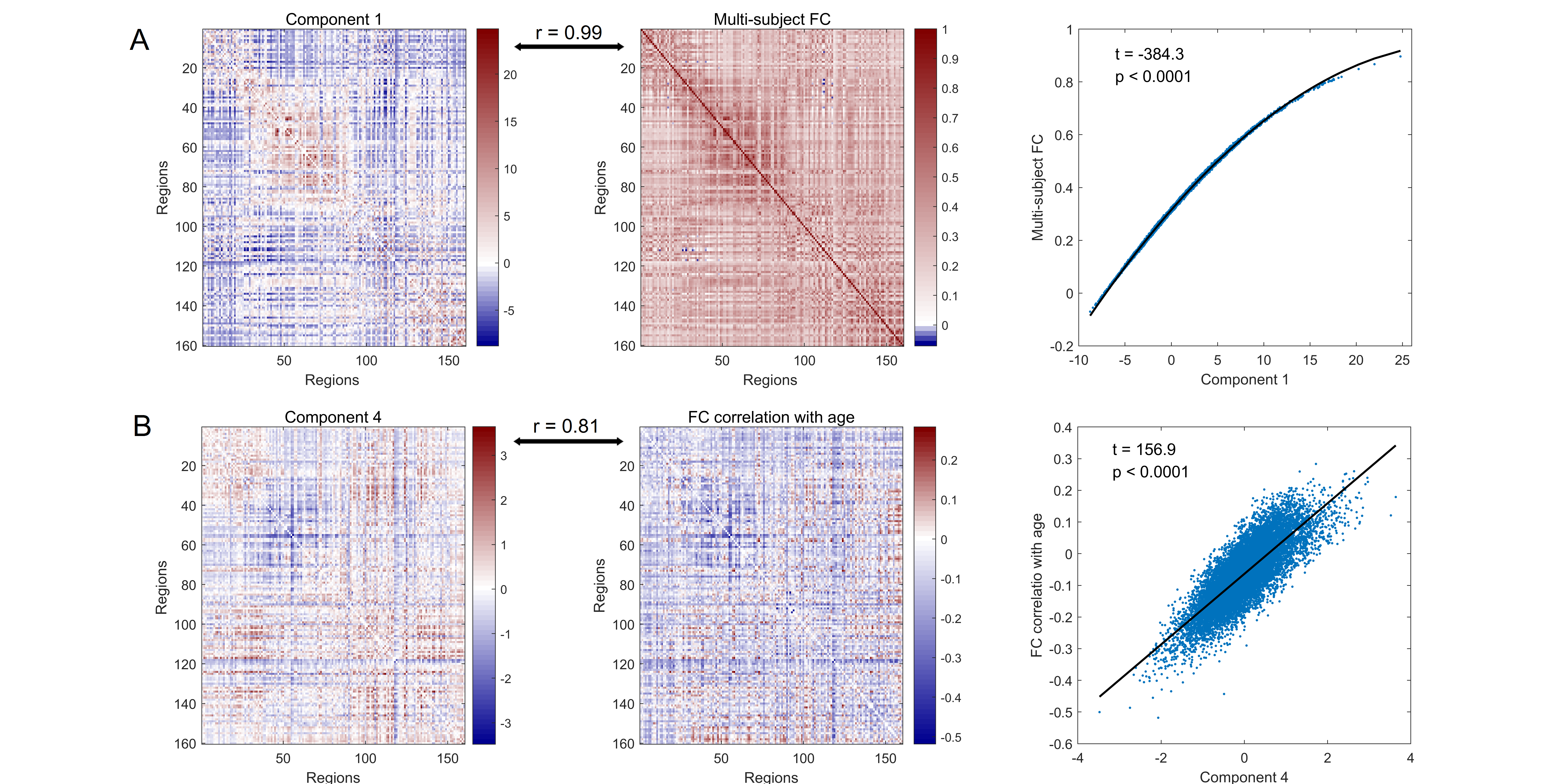

Supplement: Supplementary file 3 [file Image_2.TIF]

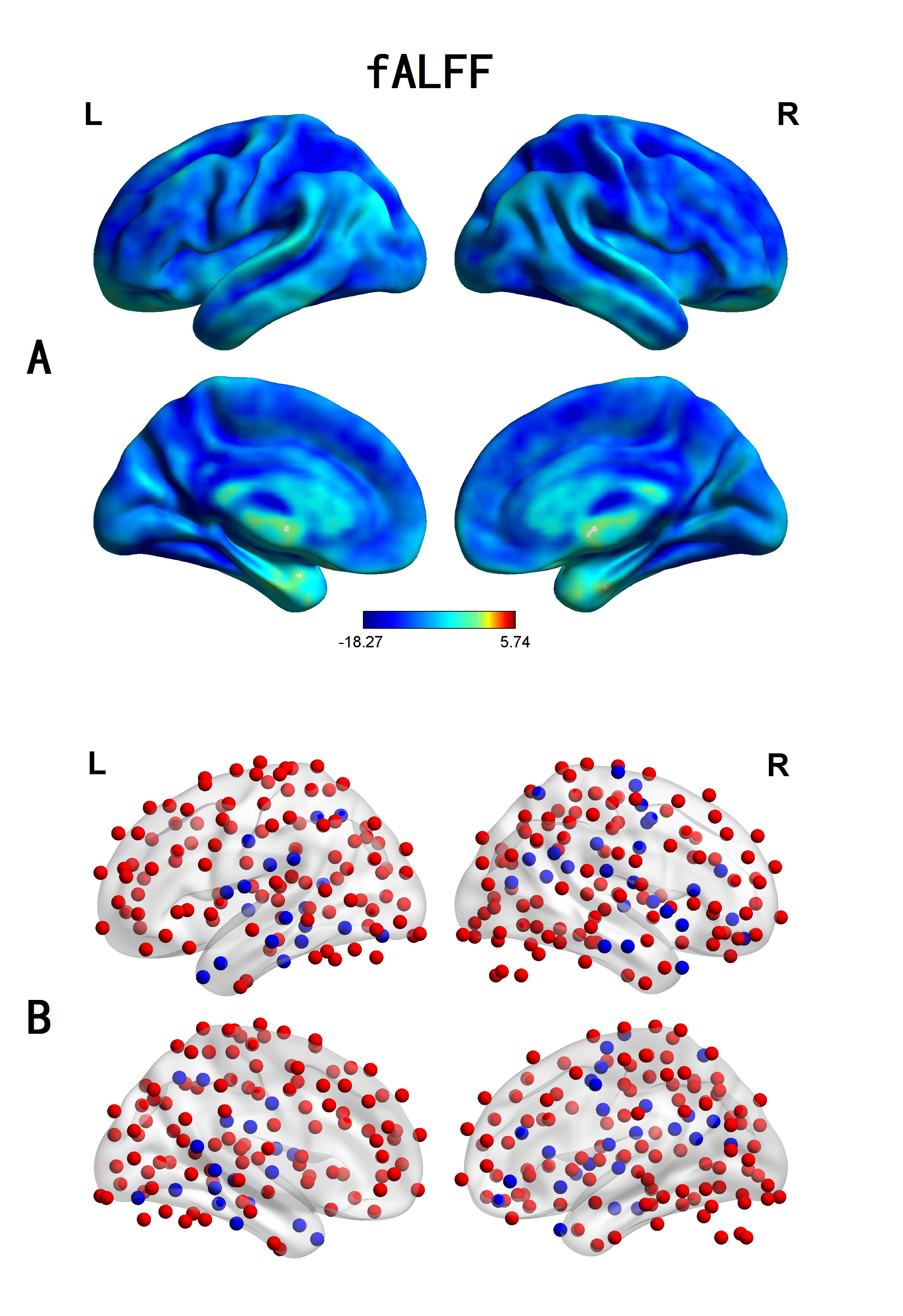

Supplement: Supplementary file 4 [file Image_3.TIF]

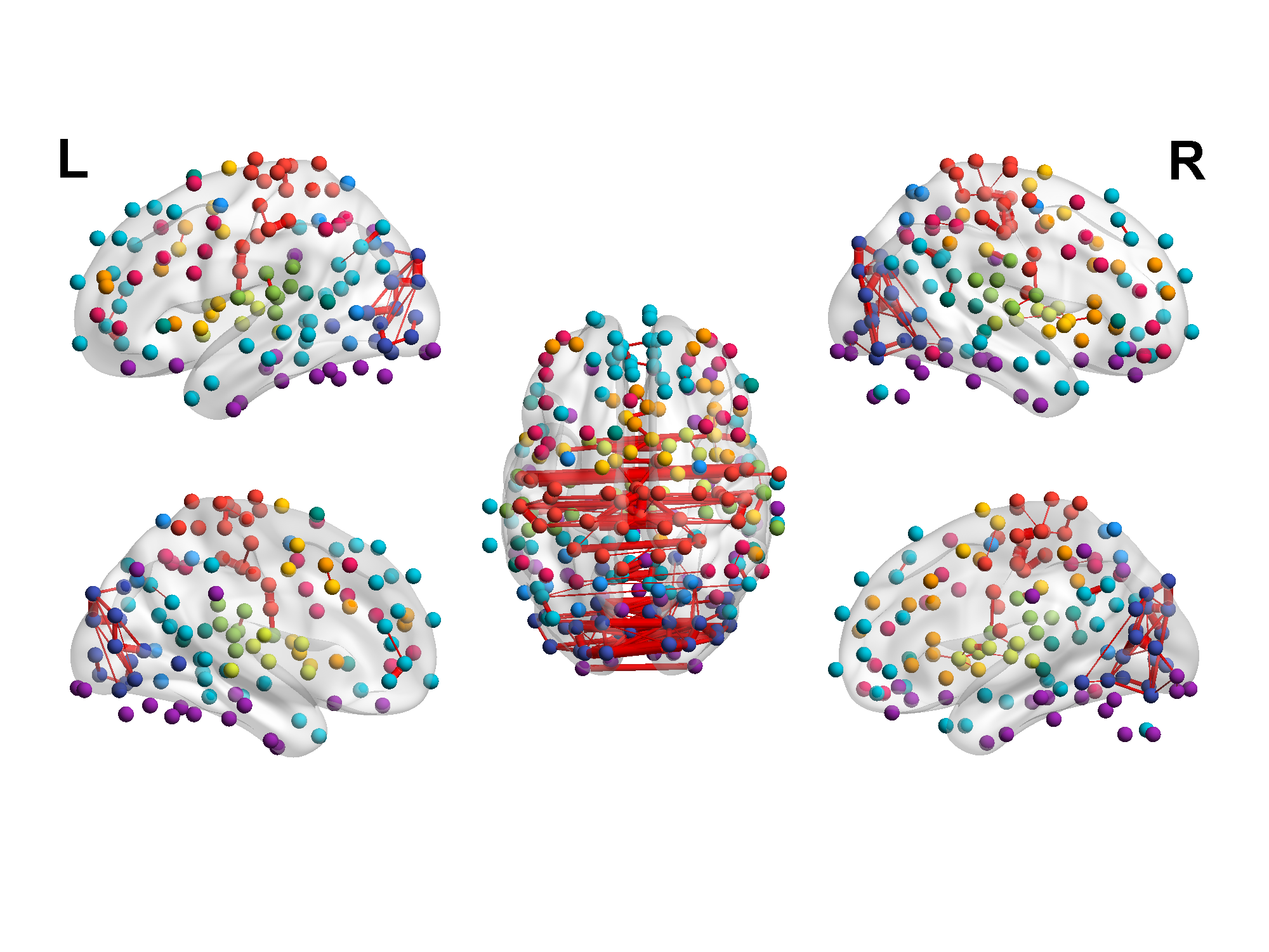

Supplement: Supplementary file 5 [file Image_4.TIF]

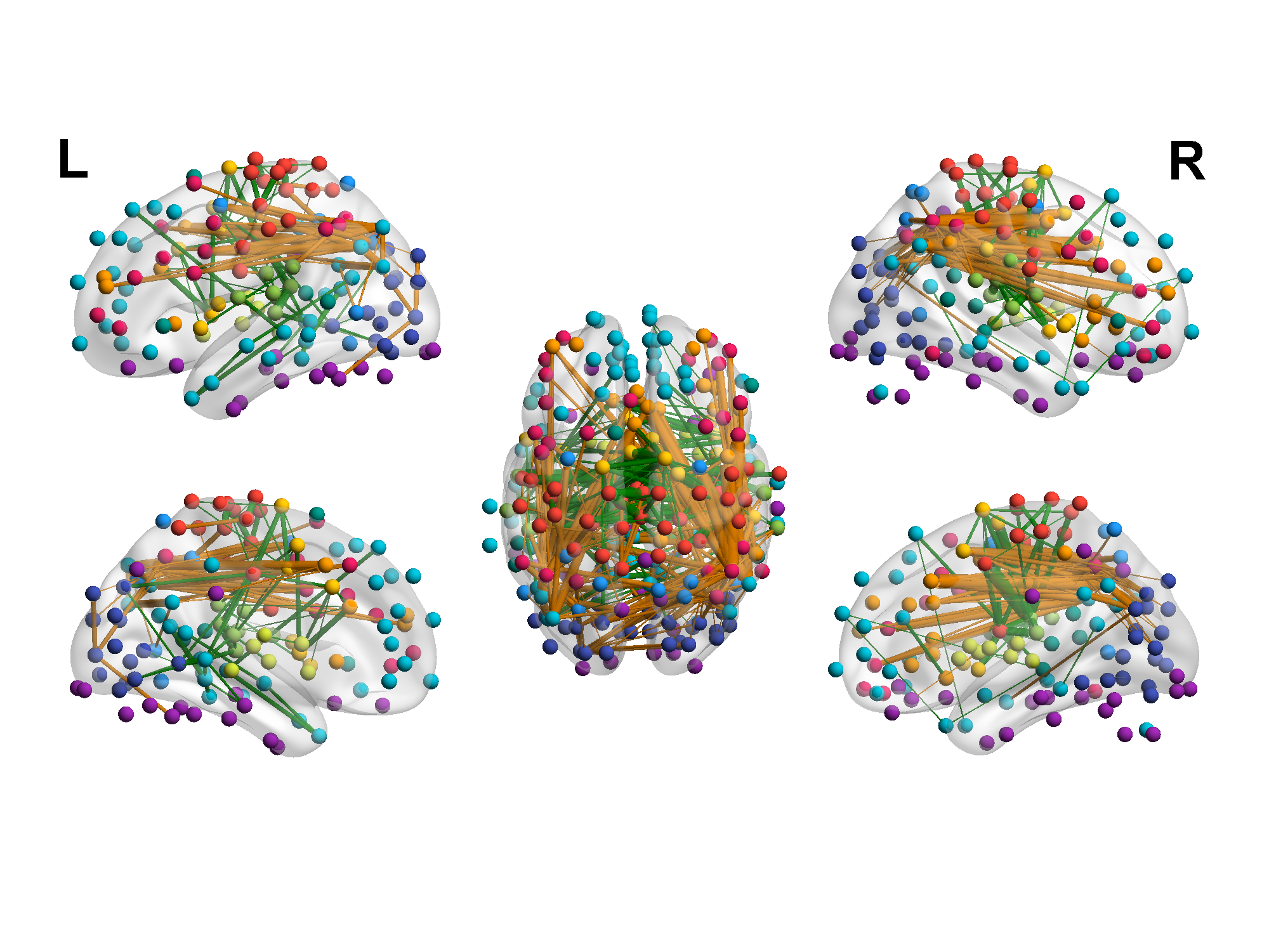

Supplement: Supplementary file 6 [file Image_5.TIF]

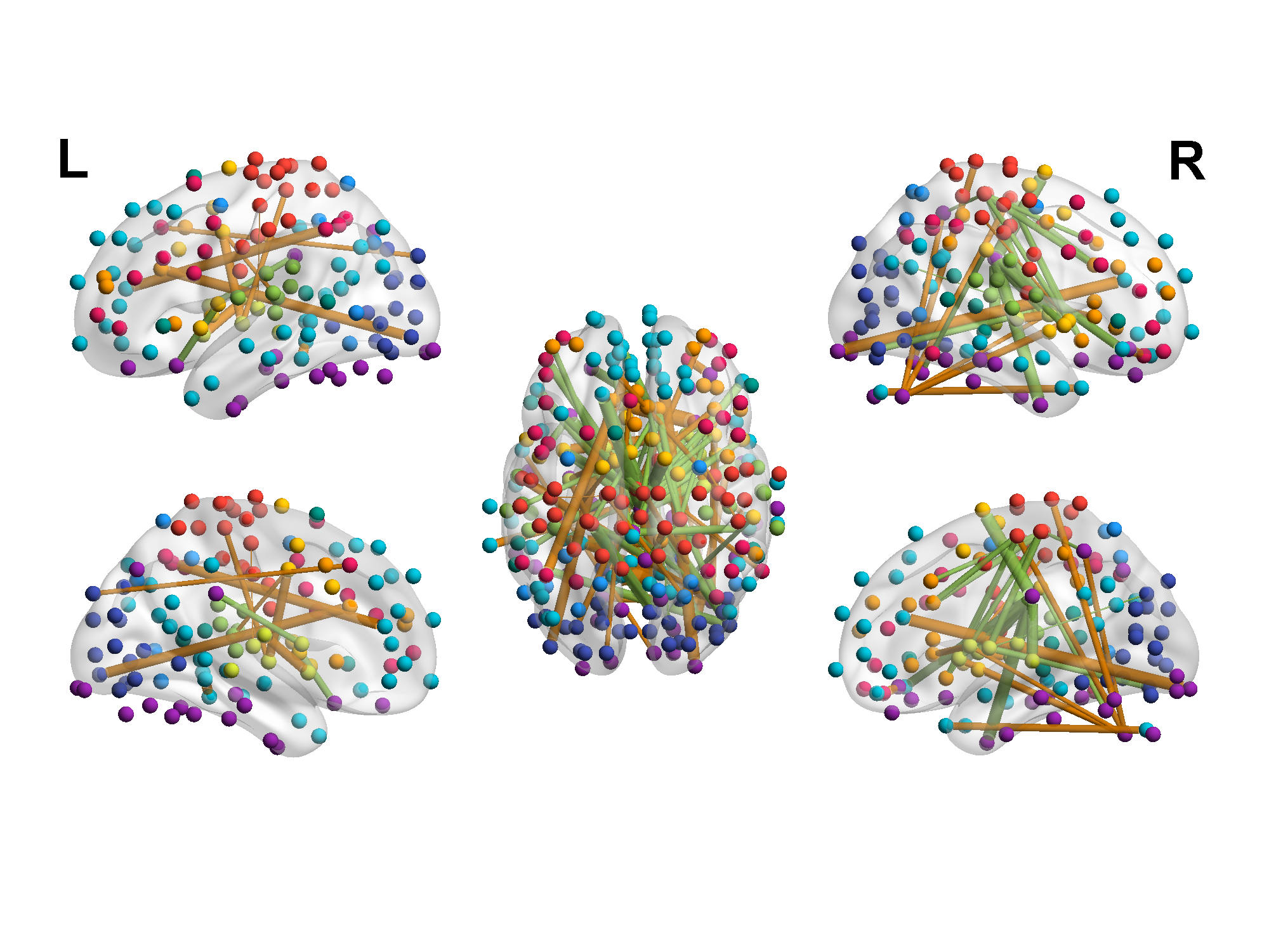

Supplement: Supplementary file 7 [file Image_6.TIF]

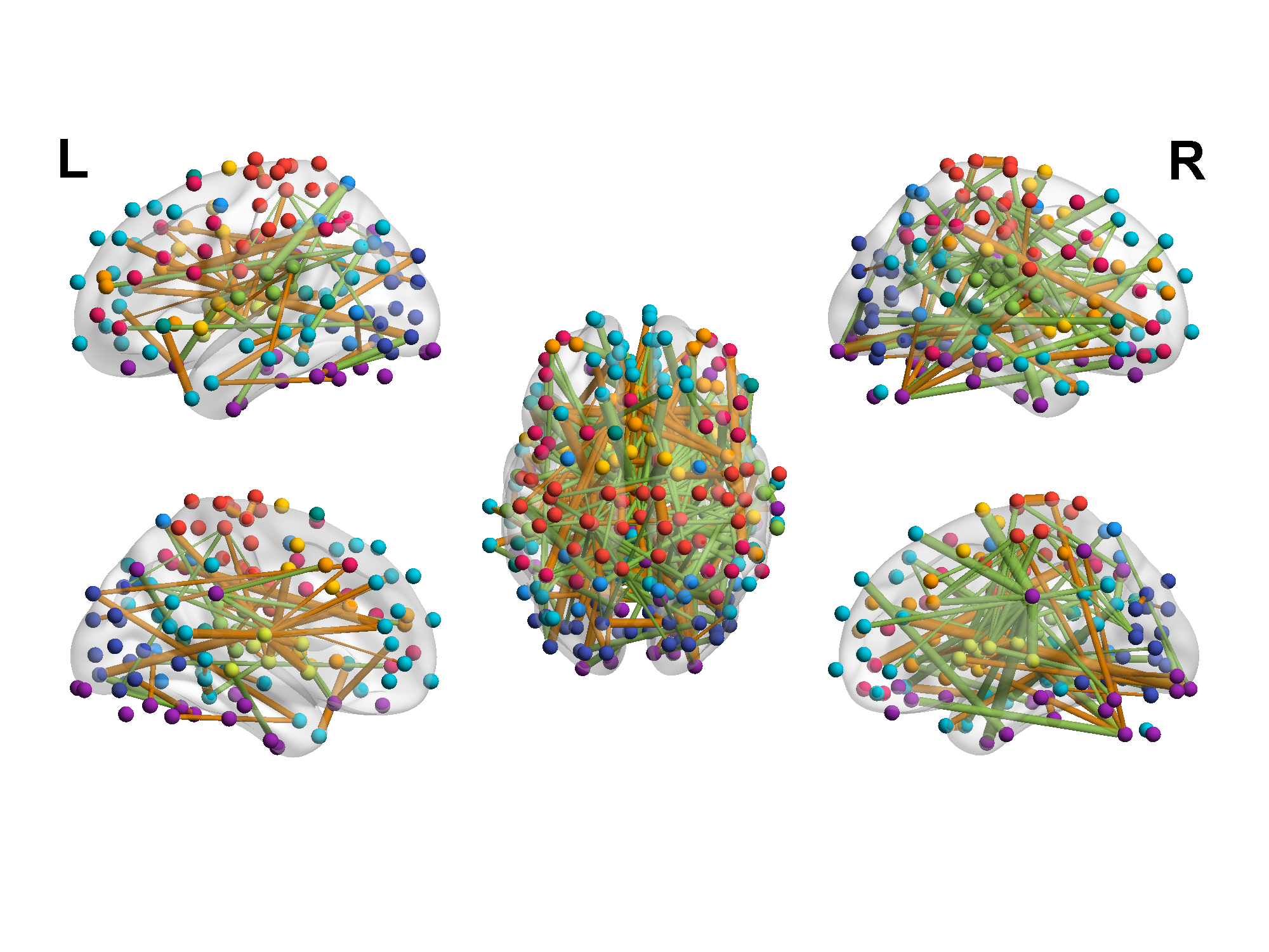

Supplement: Supplementary file 8 [file Image_7.TIF]

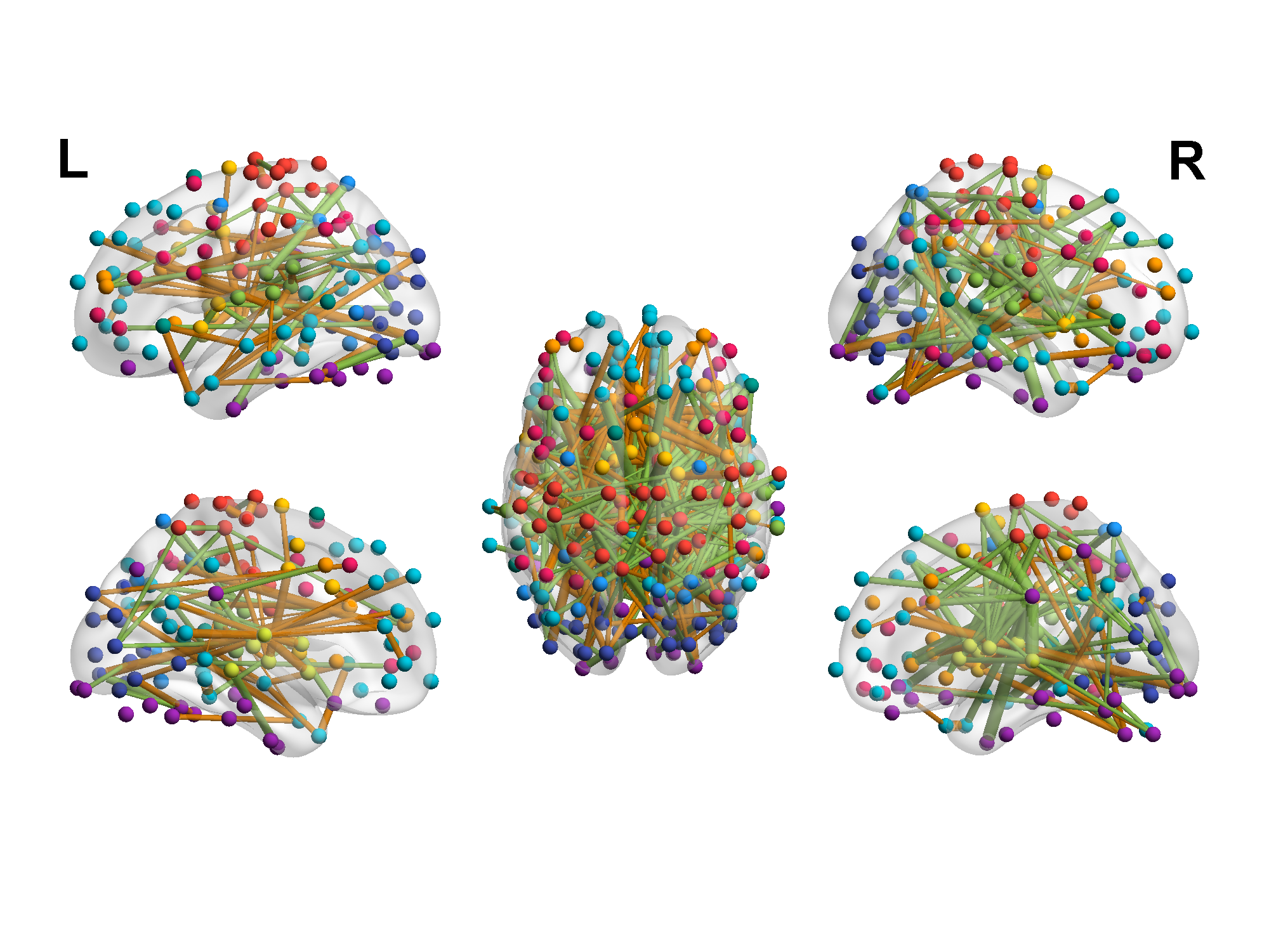

Supplement: Supplementary file 9 [file Image_8.TIF]

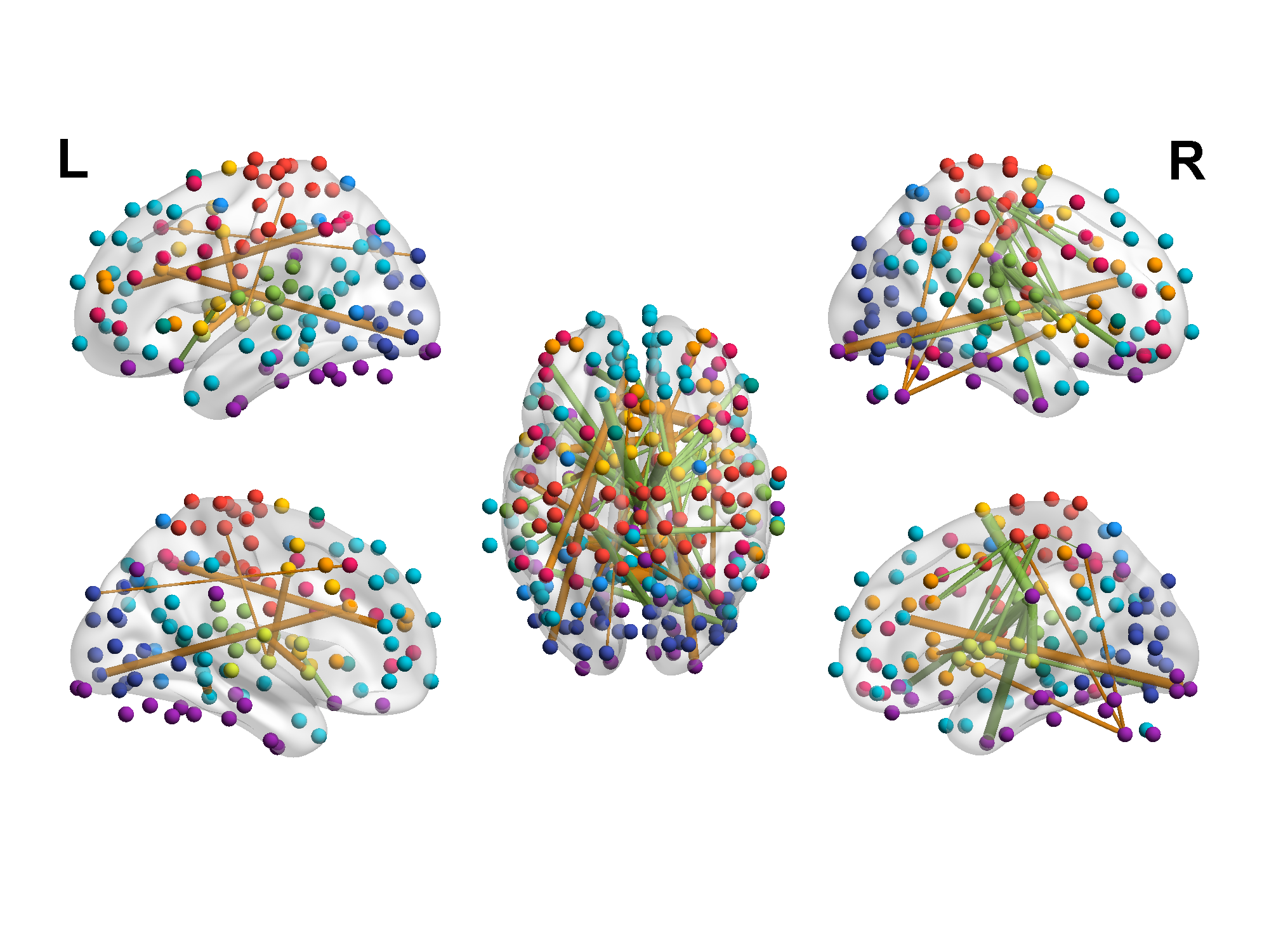

Supplement: Supplementary file 10 [file Image_9.TIF]
